# Rocky Planets as Heat Engines

**DOI:** 10.1007/s11214-026-01317-3

**Published:** 2026-08-24

**Authors:** Diogo L. Lourenço, Doris Breuer, Maëlis Arnould, Philipp Baumeister, Emeline Bolmont, Paul K. Byrne, Peter A. Cawood, Nicolas Coltice, João C. Duarte, Brad Foley, Taras Gerya, Shun-ichiro Karato, Francesca Miozzi, Lena Noack, Alexandre Revol, Tilman Spohn, Robert J. Stern

**Affiliations:** 1 Institute of Geophysics, Department of Earth and Planetary Sciences, ETH Zurich, Sonneggstrasse 5, Zurich, CH-8092 Switzerland; 2 DLR, Institute of Space Research, Rutherfordstrasse 2, Berlin, D-12489 Germany; 3 LGL-TPE, Lyon 1 Université, ENSL, UJM, CNRS UMR 5267, 2 rue Raphaël Dubois, Villeurbanne Cedex, 69622 France; 4 Department of Earth Sciences, Freie Universität Berlin, Malteserstr. 74-100, Berlin, 12249 Germany; 5 Observatoire de Genève, Université de Genève, Chemin Pegasi 51, Versoix, 1290 Switzerland; 6 Centre sur la Vie dans l’Univers, Université de Genève, Quai Ernest-Ansermet 30, Genève, 1205 Switzerland; 7 Department of Earth, Environmental, and Planetary Sciences, Washington University in St. Louis, 1 Brookings Drive, St. Louis, MO 63130-4899 USA; 8 McDonnell Center for the Space Sciences, Washington University in St. Louis, 1 Brookings Drive, St. Louis, MO 63130-4899 USA; 9 School of Earth, Atmosphere and Environment, Monash University, 9 Rainforest Walk, Melbourne, Clayton VIC 3800 Australia; 10 UMR GéoAzur, Université Côte d’Azur, 250 rue Albert Einstein, Valbonne, 06560 France; 11 Instituto Dom Luiz (IDL) and Department of Earth Sciences and Energy, Faculty of Sciences, University of Lisbon, Campo Grande, Lisbon, 1749-016 Portugal; 12 Department of Geosciences, The Pennsylvania State University, 116 Deike Building, University Park, PA 16802 USA; 13 Department of Earth and Planetary Sciences, Yale University, 210 Whitney Avenue, New Haven, CT 06520 USA; 14 Earth and Planets Laboratory, Carnegie Institution for Science, 5241 Broad Branch Rd NW, Washington, DC 20015 USA; 15 Present Address: Department of Earth and Planetary Sciences, ETH Zürich, Zürich, Switzerland; 16 Department of Earth and Environmental Sciences, University of Pavia, Pavia, Italy; 17 Department of Sustainable Earth Systems Science, University of Texas at Dallas, 800 W. Campbell Rd., Richardson, TX 75083-0688 USA

**Keywords:** Rocky planets, Heat engines, Habitability, Planetary evolution, Planetary dynamics, Magnetic field

## Abstract

Rocky planets act as heat engines, using internal heat to drive mantle convection, tectonic activity, crustal evolution and magnetic field generation. This ultimately shapes the surface environment and long-term habitability. This article reviews the physical and chemical processes governing the interior dynamics of rocky planets in the Solar System and beyond, emphasizing how variations in heat sources, material properties, internal structure, and boundary conditions (e.g., surface temperature and stellar flux) lead to diverse evolutionary pathways. Planetary heat arises from primordial accretion, core formation, radiogenic decay, tidal dissipation, and impact heating, and these energy sources regulate convective vigor and heat loss over geological timescales. Depending on different mechanical, thermal, and compositional controls, rocky planets can exhibit a spectrum of tectonic regimes, including plate tectonics, episodic lid, stagnant lid, and plutonic–squishy lid. Particular attention is given to the role of crustal buoyancy, volatile content, rheology, surface temperature, and magmatic intrusion–extrusion balance in governing lithospheric mobility and recycling efficiency. We further review models of coupled thermal and chemical evolution, including magma ocean solidification, mantle differentiation, crust formation, and volatile cycling between the interior and atmosphere. The implications of interior evolution for planetary magnetic fields are discussed, highlighting how mantle cooling histories influence dynamo generation and longevity. Finally, we review the links between tectonic regime, atmospheric evolution, and planetary habitability, with Earth serving as a reference case within a broader comparative planetary framework. By integrating insights from geodynamics, mineral physics, planetary science, and exoplanet observations, this article aims to provide a unifying framework for interpreting the interior state, thermal evolution, and tectonic diversity of rocky exoplanets, and to support future efforts to understand their dynamics, evolution, and potential for habitability.

## Introduction

The study of rocky planets has undergone a paradigm shift in the last two decades, driven by the discovery of thousands of exoplanets and by the growing understanding that many of them are terrestrial, that is, primarily composed of an iron-rich core and a silicate mantle and crust (Baumeister et al. 2025, this collection). The study of exoplanets has provided a powerful new perspective on Earth and the other rocky planets in our Solar System. By placing them within a broader planetary population, it enables a comparative perspective and allows us to start assessing whether Earth is common or exceptional. In turn, detailed investigations of rocky planets in the Solar System inform our interpretation of exoplanet observations (e.g., Byrne et al. 2026, this collection), while exoplanet studies deepen our understanding of Earth and its planetary neighbors (e.g., Spohn et al. 2026, this collection).

The processes that govern the interior dynamics, heat loss, and crustal and magnetic evolution of a planet fundamentally control its long-term surface conditions and atmosphere. Consequently, understanding the internal dynamics and evolution of rocky planets is central to assessing their potential habitability. A dynamic planet may be viewed as a heat engine: internal heat drives convection, where hot material rises, cools, and sinks, performing mechanical work that can possibly enable plate tectonics (like on Earth), sustain magnetic field generation, and/or reshape the planetary surface. The efficiency and expression of this heat engine can vary widely between rocky planets due to differences in internal structure, heat sources, rock rheology, and boundary conditions such as surface temperature, leading to different dynamical regimes (Lenardic et al. 2008; Foley et al. 2012). Convection operating within these distinct dynamical regimes can result in fundamentally different mechanisms of planetary heat loss (Turcotte 1989; Labrosse and Jaupart 2007; Moore and Webb 2013; Ricard et al. 2014; Lourenço et al. 2018; Thiriet et al. 2019) and styles of surface tectonic activity (Moresi and Solomatov 1998; Tackley 2000a; Lourenço et al. 2020). These differences, in turn, strongly influence the thermal evolution of rocky planet interiors, the temporal evolution of magmatism, the exchange of volatiles between the interior and the atmosphere, the generation and longevity of magnetic fields, and ultimately the capacity of a planet to develop and sustain a habitable surface environment.

The diversity of rocky planet evolution is already evident within our Solar System. The Moon and Mercury have old, heavily cratered surfaces and were primarily tectonically and volcanically active early in their histories. Mercury still has a magnetic field today, while the Moon’s dynamo stopped in its early evolution. Venus, on the other hand, has a geologically young surface, with estimated ages between 300 Ma and 1 Ga, relatively few impact craters, possible recent volcanic activity, a dense atmosphere, and high surface temperatures, yet it lacks a present-day internally generated magnetic field. Earth has active plate tectonics, which has produced both continental and oceanic crusts. It has a long-lived dynamo, hosts surface oceans, and is the only known inhabited planet. Mars displays a pronounced hemispheric crustal dichotomy formed several billion years ago, characterized by an ancient southern crust and a thinner, relatively younger northern crust. Like the Moon, Mars lacks a present-day magnetic field, but had one until approximately 3.7 Gyr ago. Together, the terrestrial planets and the Moon demonstrate the remarkable range of evolutionary pathways and current states that can arise even within a single planetary system (Breuer and Moore 2015). This diversity is further expanded by considering the moons of the gas giants. Jupiter’s moon Io is subject to extreme tidal heating and is the most volcanically active body in the Solar System, while Ganymede, Callisto, and Europa, with their thick ice shells and possible subsurface oceans, show additional possible interior structures and evolutionary trajectories, including environments with potential habitability. Our Solar System therefore serves as a natural laboratory for exploring possible evolutionary paths relevant to exoplanets, where an even broader diversity in size, composition, atmospheres, magnetic fields, and distance from the star is expected. For a comprehensive overview of observations of bodies in the Solar System, the reader is referred to Byrne et al. (2026, this collection).

The dynamics of a planet’s interior are therefore central to shaping its surface conditions and, looking ahead, to the interpretation and characterization of exoplanets, as they regulate key observable properties such as surface environments and atmospheric composition and play a fundamental role in determining the habitability of the planet. To better understand these relationships, this article provides an overview of variations in planetary heat engines as we currently understand them and discusses how these may manifest in exoplanets.

Section 2 outlines the physical basis for convection in planetary interiors and reviews the principal heat sources that drive convection in rocky planets. Particular emphasis is placed on the range of tectonic regimes that arise from interior dynamics, including stagnant-lid, episodic-lid, and plate-tectonic regimes, as well as regimes dominated by magmatism, such as the heat-pipe and squishy-lid regimes. Section 3 then describes the coupled thermal and chemical evolution of terrestrial planets, including crust formation and redistribution of basalt/eclogite within the interior under different regimes.

In Sect. 4, we discuss the generation and evolution of planetary magnetic fields and their dependence on the operation of the planet’s heat engine over time. In Sect. 5, we discuss the influence of the tectonic regime on habitability, bioactivity, biomass, and bioevolution, with particular emphasis on Earth as a planet operating in the plate-tectonic regime. The article concludes with a synthesis of key points and an outlook on future research directions aimed at improving our understanding of rocky planets in the Solar System and beyond.

## Interior Convection and Tectonic Regimes

### Why Does a Planetary Interior Convect?

Density variations within a fluid can drive flow when acted upon by gravity, giving rise to convection. Higher density fluid overlying lower density fluid will tend to sink, while the lower density fluid rises, ultimately driving an organized system of fluid circulation. Density variations can arise from differences in temperature or composition, though temperature variations are the primary driver for planetary interiors (e.g. Bercovici et al. 2015). Convection in the liquid iron outer core of the Earth is an exception where compositional density variations are the primary driver (e.g., Gubbins et al. 1977; Loper 1978; Lister and Buffett 1995) or potentially in early planetary evolution when a magma ocean crystallized and resulted in a chemically layered mantle (e.g., Elkins-Tanton 2008). Higher temperatures typically decrease density as a result of thermal expansion, while lower temperatures typically increase density, such that warmer fluid rises and colder fluid sinks in standard convecting systems. Convection is commonly seen in liquids and gases. Earth’s troposphere is typically undergoing convection, as the surface radiates heat absorbed from the sun, warming air near the ground and causing it to rise under its positive buoyancy. On hot and humid days, this warm air rises vigorously, creating thunderstorms. Stars also experience convection in their outer layers, creating the granulations visible on the Sun’s surface. Earth’s liquid outer core convection generates the magnetic field.

However, convection can also take place in solids that deform like fluids on geologic time scales (>∼1 Myr) at high temperature and pressure. Convection therefore occurs in the solid mantles of rocky planets. Solids flow by solid-state creep mechanisms involving dislocation motion, atomic diffusion, and grain-boundary processes, which accommodate ductile deformation under differential stress (Gordon 1965).

The heat driving convection in planetary interiors comes from many sources, such as primordial heat, radiogenic heat, latent heat, and tidal heat, reviewed in more detail below (Sect. 2.1.1). The formation of a planet is an intense process of accretion and differentiation, which leads to an initially hot interior. The energy build-up begins early during planetesimal formation (e.g., Ricard et al. 2017) and later with large impacts (e.g., de Vries et al. 2016). This initial energy from formation directly heats the planetary interior and also leads to melting and iron-silicate differentiation, the separation of rocky planets into silicate mantles and iron cores. Differentiation releases gravitational potential energy as heat, further warming the interiors of rocky planets during their very early stages. Hence, the state of a planet early on is largely molten, a state referred to as a “magma ocean.” In the magma ocean phase, the silicate mantle behaves as a liquid and will experience rapid convection and cooling, so long as the interior heat can be lost through the atmosphere to space (Monteux et al. 2016). After ∼1−100 Myrs, the mantle has mostly solidified to the point where it now behaves as a solid. Convection still operates after this but at a much slower pace as the mantle then flows via solid state creep mechanisms.

Whether convection occurs in a planetary interior (or any fluid layer) is governed by the Rayleigh number, which quantifies the competition between buoyancy forces and the combined effects of thermal diffusion and viscosity:

1 $$ Ra = \frac{\rho g \alpha \Delta T h^{3}}{\kappa \eta}, $$

where ρ is the reference density of the planetary interior, g the acceleration of gravity, α the thermal expansivity, ΔT the reference temperature contrast within the planet, h the depth of the layer considered (the mantle here), κ the thermal diffusivity, and η the viscosity, which is strongly dependent on temperature (T) and pressure (p):

2 $$ \eta (T,p) = \eta _{0} \exp \left ( \frac{E + p V}{ R T } \right ), $$

where R is the universal gas constant, E and V are, respectively, the activation energy and the activation volume, and $\eta _{0}$ is a pre-factor that depends on the stress, composition and grain-size. Viscosity variations within a planetary mantle are expected to span more than 10 orders of magnitude, making it difficult to fully represent the flow behavior of rocks throughout the planet’s interior using the reference viscosity η in equation (1). If Ra is larger than a critical value (in general close to 1000), convection occurs. As Ra increases, the vigor of convection intensifies, indicating a greater potential for cooling.

A fundamental property for convection to operate in a fluid is its rheology, which is particularly complex in rocks because they exhibit elastic, viscous, and plastic behavior, encompassing a wide range of deformation mechanisms (Burov 2011; Karato 2010), and may be further influenced by compositional variations and partial melting. Despite this complexity, in particular regarding mantle viscosity, it is nevertheless possible to obtain first-order estimates of the Rayleigh number for Earth and other terrestrial planets. Constraints on the average viscosity of the Earth’s upper mantle are provided by observations of post-glacial rebound following ice sheet retreat after the last glacial maximum, resulting in values of $\eta \sim 10^{21}-10^{22}$ Pas (e.g., Mitrovica and Forte 1997). Adopting representative mantle parameters of average mantle density $\rho \approx 4500\text{ kg/m}^{3}$, $g=10\text{ m/s}$, $\alpha = 3 \times 10^{-5}$ 1/K, $\Delta T \approx 1350\text{ K}$, $h \approx 2890\text{ km}$, and $\kappa \approx 10^{-6}\text{ m}^{2}\mathrm{/s}$, the resulting Rayleigh number for Earth’s mantle is $Ra \approx 4 \times 10^{6}-4\times 10^{7}$. This value exceeds the critical Rayleigh number by several orders of magnitude, indicating that the Earth’s mantle is vigorously convecting. Comparable first-order estimates can be made for the other rocky planets in the Solar System as well as for rocky exoplanets. The mantles of the terrestrial planets and the Moon are therefore very likely still convecting today, with the possible exception of Mercury, whose thin mantle may inhibit convection (Jain and Solomatov 2024). Rocky exoplanets would need drastically different properties (e.g., mantle viscosity 3–4 orders of magnitude larger than Earth’s or a thin mantle such as Mercury) to not also have convecting mantles.

Furthermore, the interiors of the terrestrial planets have cooled substantially since their formation and early evolution. Higher mantle and/or core temperatures in the past and potentially higher volatile contents would have further increased the Rayleigh number, implying that mantle convection was likely even more vigorous earlier on (see Sect. 2.3.1 for more details). Elevated mantle temperatures increase the temperature contrast ΔT in Eq. (1) and simultaneously decrease the viscosity of the mantle η, since the deformation mechanisms governing the viscous flow in silicate rocks are thermally activated and become more efficient at higher temperatures. Consequently, present-day conditions on the planets are not uniquely optimized for the operation of mantle convection. Rather, convection is a natural outcome of the intense heat associated with planet formation and of internal heat sources that remain active today, as discussed below. Mantle convection is therefore expected to be a common mechanism in the silicate mantles of rocky planets.

#### Heat Sources

The heat that powers the planetary heat engine has different sources. Some sources dominate during early planetary evolution, including primordial heat retained from accretion and energy released during core formation. Others become increasingly important at later stages, such as radiogenic heating and energy input from impacts occurring after the main accretion phase. Additional processes, such as tidal heating caused by proximity to a host star or a much more massive planet, may contribute during both early evolution and later times.

1. **Primordial heat** In the early thermal evolution of rocky planets, heat is produced by two main processes: accretion and core formation. During accretion, kinetic energy from the collisions of planetesimals is converted into thermal energy, heating the growing planetary body. As the body grows, the energy input increases, resulting in a temperature profile with lower temperatures in the center and increasing temperatures towards the surface. As a result, local magma ponds or global magma oceans can form when the melting temperature of the material is exceeded. This is more likely the larger the growing body. It is assumed that the inversion of the temperature profile, with the more common profile of a hot interior and decreasing temperature toward the surface, occurs very quickly and is associated with rapid core formation. This involves the separation and sinking of dense materials (mainly iron and nickel) within the molten planetary mantle to form a metallic core. As these materials sink, they release gravitational potential energy, which is converted into thermal energy and further contributes to the heating of the planet.
2. **Radiogenic heating** Radioactive heating involves the decay of radioactive isotopes within the planet’s interior, which releases energy in the form of heat. The primary radioactive isotopes with short-half lives responsible for radiogenic heating are ^26^Al and ^60^Fe, and with long half-lives are ^235^U, ^238^U, ^232^Th, and ^40^K. Short-lived radionuclides dominated the thermal evolution of planetesimals that formed very early, within <∼2 Myr after the formation of Calcium–Aluminium-rich inclusions (CAIs), the oldest dated solids in the Solar System. Their decay provided sufficient energy to drive extensive melting and differentiation, implying that many planetesimals contributing to planet formation were already differentiated into iron cores and silicate mantles. In contrast, long-lived radionuclides primarily contribute to a planet’s internal heat budget after accretion and core formation and represent a major ongoing power source for the planetary heat engine. For Earth, the contribution of the radioactive heat in the mantle and crust to today’s surface heat flow (also called Urey ratio) is about 35–50% (Korenaga 2008; Gando et al. 2011); the rest, known as secondary cooling, is mostly stored as primordial energy. For Mars, the Urey ratio is estimated to be higher, at approximately 60% (Plesa et al. 2015).
  Radiogenic heat sources are distributed unevenly within a planet’s interior. The crust is typically enriched in heat-producing elements by a factor of 5–15 relative to the mantle, with continental or $SiO_{2}$-rich crust more enriched than oceanic crust (e.g., Turcotte and Schubert 2014). As crust is formed by partial melting of the mantle over time, radioactive elements are redistributed from the mantle into the crust. The abundance of radiogenic heat sources in planetary cores, however, remains a subject of active debate. Because these elements tend to be lithophile and do not go into the iron melt, it is usually assumed that there is only a small amount of radiogenic heat sources in the core. However, U and Th can become siderophile (iron-loving) and enter the iron melt under highly reducing and sulfur-rich conditions (Wohlers and Wood 2015). In addition, at the high pressures expected in super-Earth interiors, the metal–silicate partition coefficients of radioactive elements may change substantially. Using ab initio molecular dynamics simulations, Luo et al. (2024) demonstrated that the cores of large rocky planets may store significant amounts of radiogenic heat, with important consequences for their long-term thermal evolution and the generation and sustainability of planetary magnetic fields.
3. **Tidal heating** Tidal heating can be an important heat source for planets or satellites, depending on their orbital and rotational configurations and internal properties. Rocky planets and satellites are viscoelastic bodies in which deformation energy - such as that from cyclic tidal deformation - is dissipated as heat. The viscoelastic properties can be represented by a complex modulus, $\tilde{\nu}$, which depends on the elasticity, the viscosity, and the period of the tidal deformation. These properties can be visualized by combinations of purely elastic springs and purely viscous dashpots. A simple model that is often used is the Maxwell rheology, in which the spring and the dashpot are connected in series. The complex modulus $\tilde{\nu}$, is then given by $\tilde{\nu} = i\omega _{T}\nu /(i\omega _{T} + \nu /\eta *)$, where ν is the shear modulus, η the viscosity and $\omega _{T}=2\pi /T_{T}$, the period of the tidal deformation. The frequency at which the dissipation rate is maximum is given by the ratio of the shear modulus to the viscosity, which is the reciprocal of the Maxwell time. The dissipation rate diminishes rapidly for forcing periods much smaller or much larger than the Maxwell time.
  A rheological model that is increasingly used in recent work is the Andrade rheology (e.g., Tobie et al. 2019; Bolmont et al. 2020). Variants of the model have been discussed by Renaud and Henning (2018), Walterová et al. (2023), and Bierson (2024). A major difference is the addition of a time-dependent spring-dashpot element to the Maxwell series or, in some cases, further combinations of springs and dashpots. One characteristic variant of this model, as discussed by Bierson (2024), has $1/\tilde{\nu} = 1/\nu - i/\omega _{T}\eta + \beta (i\omega _{T})^{- \alpha} + \Gamma (1+\alpha )$, where α and β are empirical constants and Γ is the gamma function. The Andrade rheology exhibits weaker frequency and viscosity dependence in the viscoelastic response as compared to the Maxwell model. It better fits laboratory observations (Henning and Hurford 2014) and compares favorably with the viscoelastic properties of the Earth (Tobie et al. 2019). The Andrade rheology would allow for significant tidal heating at lower mantle temperatures and thus higher viscosities than the Maxwell rheology.
  In order for deformation energy to be continously dissipated, the tidal deformation must vary periodically with time. Therefore, for a planet or satellite in a circular orbit with no obliquity of the rotational axis, tidal dissipation requires that the rotational angular velocity, ω, be different from the mean orbital angular velocity, n. In cases of synchronous rotation, when ω=n, the orbit must be eccentric and/or the rotation axis must be tilted relative to the orbital plane. Viscoelasticity causes a phase lag between the tidal bulge and the line connecting the centers of mass of the central and orbiting bodies. This phase lag is a measure of the tidal dissipation rate. Since dissipated energy comes from the planet’s or satellite’s orbital and rotational energy, tidal dissipation tends to reduce these factors, thus leading to synchronous rotation, decreasing obliquities, and expanding orbits with decreasing orbit eccentricities,^1^ unless the planet or satellite is caught in resonances.
  The most prominent examples of tidally heated bodies in the Solar System are Io and Enceladus. Io is a satellite of Jupiter that is only slightly larger than the Moon. Its rocky mantle is heated by around 100 TW of tidal power, which is two orders of magnitude greater than the radiogenic heating it would receive if its chemical composition were similar to that of the Moon (e.g., Spohn 2015; Kervazo et al. 2021). Enceladus, an icy satellite of Saturn with a diameter of 500 km, has significant cryovolcanic activity near its south pole. It is heated by 20–40 GW (e.g., Nimmo et al. 2023). Both satellites are in synchronous orbits, with small but significant eccentricities caused by orbital resonances, and insignificant obliquities. For Io, the resonance is with Europa and Ganymede; this is known as the Laplace resonance (e.g., Peale and Canup 2015). For Enceladus, the resonance is with Dione. Close-in exoplanets such as the TRAPPIST-1 planets, are potential candidates for significant tidal heating, roughly 10–20 TW for TRAPPIST-1e and f (Bolmont et al. 2020 and compare Ducrot et al. 2026, this collection).
  As demonstrated by e.g., Segatz et al. (1988), Wisdom (2008), and Beuthe (2013), the tidal dissipation rate $\dot{E}$ for rotationally symmetric, layered planets or satellites can be calculated from
  3 $$ \dot{E}=-\frac{21}{2}\mathrm{Im}(\tilde{k}_{2})\frac{(nR)^{5}}{G} f(e, \phi ) $$
  where R is the radius of the planet or satellite and G is the gravitational constant. $\mathrm{Im}(\tilde{k}_{2})$ is the imaginary part of the complex tidal Love number $\tilde{k}_{2}$. The latter is defined as the ratio between the additional potential raised by the tidal deformation to the tide raising potential (Love 1909) and depends on the interior layering and the viscoelastic properties through the complex modulus $\tilde{\nu}$ introduced above. The function f(e,ϕ) depends on the eccentricity e and the obliquity ϕ. For small values of e and ϕ, as well as synchronous rotation,
  4 $$ f(e,\phi )=e^{2}+\frac{1}{7}sin^{2} \phi $$
  (Beuthe 2013). Expressions for f(e,ϕ) for arbitrary values of e and ϕ and for non-synchronously rotating planets or satellites are given in Wisdom (2008).
  It is important to note that, for solid rocky planets and satellites, a feedback mechanism may be at work: since the viscosity and, to a lesser extent, the shear modulus are temperature dependent, heating by tidal dissipation increases temperature, decreases viscosity and increases the dissipation rate. The latter will tend towards equilibrium with the heat transfer rate, which also increases with temperature. The feedback mechanism breaks down at supersolidus temperatures, when the rheology is dominated by the viscous melt.
  Calculating $\mathrm{Im}(\tilde{k}_{2})$ can be a demanding task (e.g., Segatz et al. 1988; Tobie et al. 2019; Bolmont et al. 2020), often requiring parameter values that are difficult to estimate. Therefore, simplified models replace $\mathrm{Im}(\tilde{k}_{2})$ with $k_{2}/Q$, where $k_{2}$ is the modulus of $\tilde{k}_{2}$ and Q is a quality factor that quantifies the energy loss per tidal cycle in terms of the total deformational energy. Other models use a constant tidal time lag (Hansen 2010; Hut 1981; Bolmont et al. 2011, 2020) or phase angle (Makarov and Efroimsky 2013). These approaches simplify the dependence on frequency and temperature, but retain dependence on orbital and rotational parameters and facilitate dissipation rate estimates.
4. **Other sources of heating** Even after the main phase of accretion and magma ocean crystallisation, impact processes can significantly influence the thermal evolution of the planets. Impact events can create localized thermal anomalies that affect the planet’s surface and subsurface temperature distribution. The distribution and energy converted into heat between the impactor and the target is dependent on the mass, velocity and impact angle of the impactor. In a simple form, the total energy released during impact ($E_{impact}$) can be scaled with the mass m and the velocity v of the impacting body: $E_{impact} = \frac{1}{2} m v^{2} $. This can lead to an increase in temperature ($\Delta T \propto \frac{v^{2}}{c}$ with c the specific heat capacity) or even to melting depending on the temperature distribution before the impact and the melting temperature of the target. The melting and even vaporization of the rock during the formation of impact craters is usually attributed to shock compression. However, other mechanisms such as plastic deformation and decompression due to structural uplift also contribute to the formation of melt. Scaling laws (Watters et al. 2009) have been developed to calculate the local temperature increase in the mantle. These scaling laws determine the amount of heat deposited deep in the mantle by the shock waves of large impacts, taking into account the increasing pressure and density with depth. The influence of large and giant impacts on a planet’s thermochemical evolution, tectonic processes (e.g. the initiation of plate tectonics), atmospheric evolution, and magnetic field has been investigated in numerous studies (e.g., Ruedas and Breuer 2017; Roberts and Arkani-Hamed 2017; O’Neill et al. 2020; Borgeat and Tackley 2022; Johnson et al. 2026).
  In addition, the host stars themselves can influence the thermal regime of close-in orbiting planets. This is especially relevant for tidally locked planets, where only one side of the planet is continuously irradiated by the star, leading to a hot day-side and cooler night-side, at least in the absence of a substantial atmosphere. Depending on the distance of the planet to the star, the temperature difference between the day- and night-side can reach several hundreds to thousands Kelvin, as observed for example for 55 Cancri e (Demory et al. 2012, 2016). Thick atmospheres, however, may efficiently redistribute heat and reduce day–night temperature contrasts, similar to what is observed on Venus (Kane 2022). This process is an active area of research in the exoplanet community (e.g., Carone et al. 2018; Hammond and Lewis 2021).
  Another potential impact that a star can have on the heat budget of close-in rocky exoplanets arises from the interaction between the magnetic field of the star and the orbiting planetary body, provided the planet possesses an electrically conductive mantle (e.g., due to the presence of iron or water in the silicate mantle, particularly in the crust). Depending on the strength and alignment of the stellar magnetic field, and the orbital inclination of the planet, as well as the orbital distance of the planet from the star, such interactions could generate extreme internal ohmic heating, potentially comparable to the tidal heating observed for Io (Kislyakova et al. 2018).

### Tectonic Regimes

A tectonic regime, also called a magmatotectonic regime, can be defined as the surface expression of the interior dynamics of a planet. In this sense, the interior of a rocky planet is either convecting or has convected in the past. However, the surface expression of these dynamics differs between planets, depending on magmatic input, lithospheric strength and convective forces. In fact, Earth, which exhibits a plate tectonics regime, appears to be unique, at least today and based on the observations we have so far. It is also important to note that the tectonic regime that a planet experiences can change over time and even vary across its surface at a given time. For example, a change in Earth’s tectonic regime has been proposed (see review by Korenaga (2013)), and hemispherical tectonics has been suggested for certain exoplanets (e.g., Meier et al. (2021)). Furthermore, the active tectonic regime may also depend on the history of a planet. For example, it has been found that complex feedback mechanisms can lead to a hysteresis of states where identical physical parameters result in non-unique tectonic states (e.g., Lenardic and Crowley (2012), Weller and Lenardic (2012), Weller et al. (2015)).

**Fig. 1:**
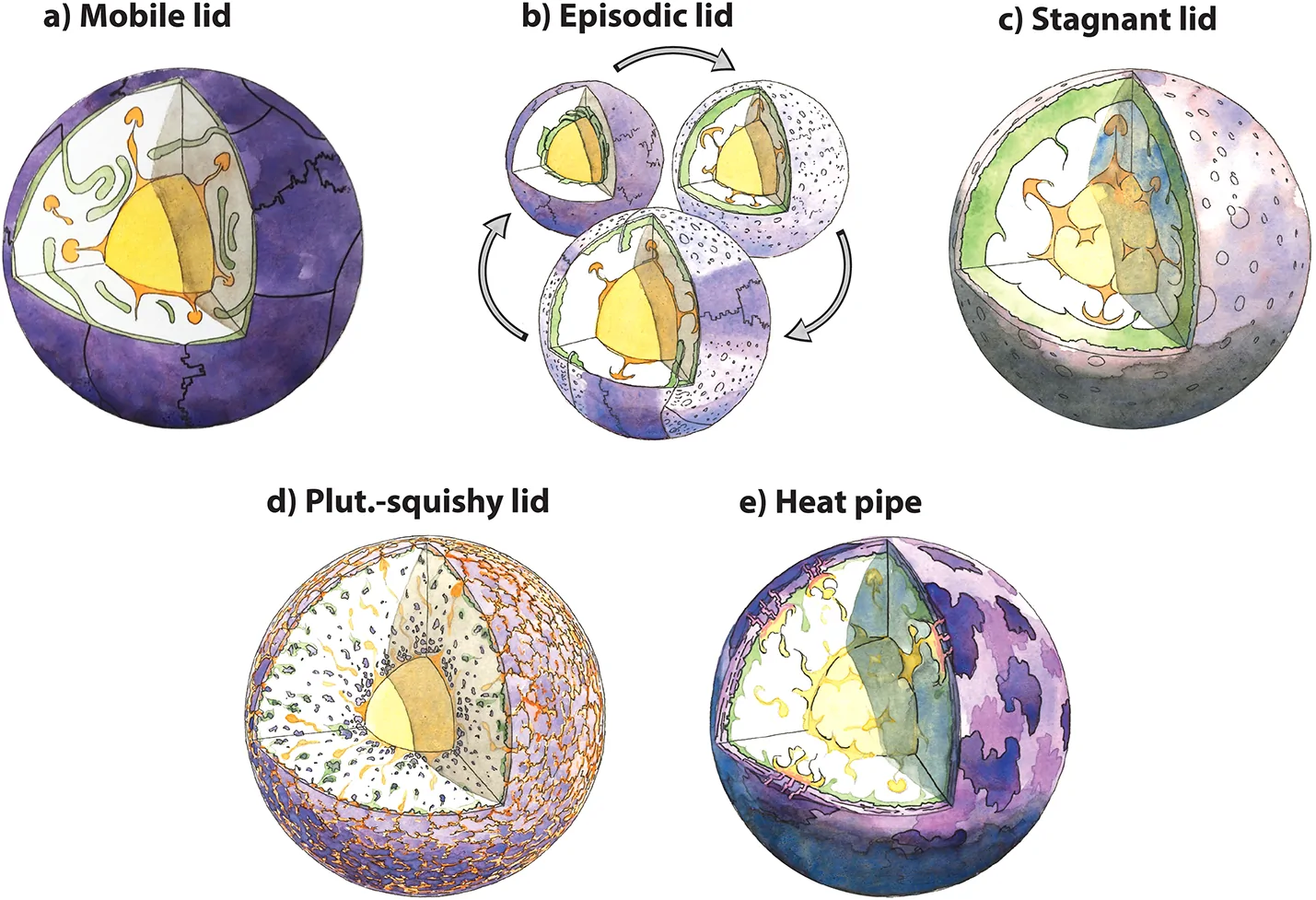
Global depictions of different tectonic regimes, showing the relation between surface and interior (lithosphere and mantle) dynamics. The core is the orange sphere in the center, and orange mantle plumes/upwellings rise from the core-mantle boundary. White material represents the ambient mantle, green represents the lithosphere, and purple-ish colors are basaltic crust. The darker the purple, the newer the crust (and therefore less cratered). Figure from Lourenço and Rozel (2023)

Next, we introduce the various tectonic regimes that have been identified (Fig. 1) and their connections to different celestial bodies. For a more detailed comparison of these tectonic regimes with observations from the Solar System, the reader is referred to Byrne et al. (2026, this collection). We note that the exploration of tectonic regimes is recent, and additional regimes may yet be identified. Furthermore, the coexistence of, and transitions between, these regimes remain poorly constrained.

#### Mobile Lid / Plate Tectonics

Plate tectonics is the regime active on modern-day Earth. It is characterized by relative surface motion between lithospheric plates, which are continuously recycled back into the mantle through subduction, a process in which oceanic lithosphere descends as a coherent slab into the mantle because of its negative buoyancy. New oceanic crust is formed at divergent plate boundaries through decompression melting of mantle material, and is the top part of the oceanic lithosphere that forms from the cooling of the mantle. The continental lithosphere, on the other hand, is thicker, chemically evolved, and more buoyant than the oceanic lithosphere and, therefore, resists subduction. Continents are long-lived, preserving geological records over billions of years, yet they are continually reshaped and drift at the surface of the Earth. Dense lower crust or lithospheric mantle may be lost through delamination or drip tectonics. Mobile lid (Fig. 1a) is generally used as the numerical equivalent of plate tectonics (e.g., Tackley 2000a; Stein et al. 2004), recognizing that numerical models are always idealized.

For rocky planets with extremely hot surfaces (e.g., ∼1000 K), where the lithosphere and crust are weakened by the hot temperatures (i.e., reduced viscosity), rather than by plasticity, the mobile (sluggish) regime may then accurately represent a convective mobilization state. This leads to resurfacing also in the absence of plate tectonics, as has been proposed for Venus during a hotter stage in its past (Noack et al. 2012).

#### Episodic Lid

An episodic lid (Fig. 1b) is characterized by surface mobility bursts due to episodic overturns of an unstable stagnant lid (Turcotte 1993; Moresi and Solomatov 1998; Armann and Tackley 2012) or internal mantle instabilities (Davies 1995; Bédard 2018). These fast and intermittent recycling episodes are separated by longer phases of stagnant lid convection. This regime has been proposed for modern-day Venus with the most recent episodic phase believed to have occurred about between 300 and 1000 Ma (Turcotte 1993), and for the Archean Earth (e.g., Bédard 2006, 2018).

#### Stagnant Lid

A stagnant lid (Fig. 1c) shows little to no evidence of surface motion, likely being covered by a thick and non-yielding lithosphere (Nataf and Richter 1982; Christensen 1984). Stagnant lid is a broad definition that can cover different types of planets: “geologically dead” planets that have lost a significant part of their energy and have no active internal processes, planets that still experience some volcanism or small-scale tectonics such as the Moon or Mars, and planets that do not exhibit any surface motion but still experience high degrees of activity, for example, in terms of volcanism (see Heat pipe).

#### Plutonic-Squishy Lid

A plutonic-squishy-lid regime (Fig. 1d) is characterized by small, strong blocks or small plates separated by warm, weak regions formed by plutonism. Near these weak zones, eclogitic (high-density rocks formed from basaltic crust at pressures of ∼2–3 GPa and higher) drippings and lithospheric delaminations drive significant surface motion toward the delamination focus, even in the absence of active subduction. Block boundaries shift over time, primarily forming in areas of magma intrusion, resulting in transient, small plates. This regime produces a thin lithosphere, leading to high conductive heat fluxes and lower upper mantle temperatures compared to a stagnant lid or even a heat pipe (Lourenço et al. 2018, 2020). It has been proposed that it could be applicable to modern-day Venus (Byrne et al. 2021b; Smrekar et al. 2023) and early Earth (Sizova et al. 2015; Fischer and Gerya 2016).

#### Heat Pipe

A heat pipe regime (Fig. 1e) is commonly characterized as a stagnant lid because there is no significant horizontal surface motion. However, in a body that experiences heat pipe, melt generated in the mantle is efficiently transported to the surface, and a thick lithosphere develops as a result of frequent volcanic eruptions that advect surface materials downwards. This vigorous volcanic activity dominates heat transport and can cool a planet efficiently (Moore et al. 2017). This regime has been proposed to be active on Jupiter’s moon Io as a consequence of strong tidal heating (e.g., Moore 2001), and it has also been suggested to be active on the early Earth (Moore and Webb 2013).

### What Controls Convection and the Active Tectonic Regime?

#### Interior Structure and Composition

1. **Introduction to possible structure and compositions**
  The interiors of planets can have a range of structures depending on their initial composition and evolutionary stage (e.g., Baumeister et al. 2025, this collection). Rocky planets start their evolution in a largely or fully molten state, during which iron–silicate separation occurs, leading to the formation of an iron-rich core and a silicate mantle. As the interior cools sufficiently, crystallization of mineral phases can take place, and the mantle can develop a layered structure. For planets that are located sufficiently far from their stars, crystallization and subsequent crystal–melt separation can produce horizontally stratified layers composed of distinct mineralogical assemblages, as well as a buoyant primary crust. Whether the mantle has reservoirs/layers of varying compositions or is relatively homogeneous depends on various factors, including the time scale of magma ocean solidification, the density of the forming phases compared to the remaining liquid, and the efficiency of mantle mixing (see Sect. 3.1). In recent years, the discovery of tidally locked planets with very short orbital periods has motivated the development of new interior models (Meier et al. 2021). In these planets, crystallization is thought to occur at the boundary between the hot and cold hemispheres, resulting in the formation of a vertically oriented crystal layer.
  The relative sizes of a planet’s major components, such as the core, mantle, and crust, play an important role in its thermal and tectonic evolution, as well as the overall planetary size (see Sect. 3.5). For example, a large ratio of core radius to mantle radius results in a relatively thin mantle, which cools more rapidly and transitions to conductive heat transport sooner. In addition, the corresponding mantle convection structure is of smaller scale than that of a planet of the same size but with a smaller core. Within our Solar System alone, core-to-mantle radius ratios vary significantly, ranging from approximately 0.8 for Mercury to about 0.2 for the Moon (e.g., Byrne et al. 2026, this collection).
  For most exoplanet applications, planets are commonly considered as layered spheres in hydrostatic equilibrium. The center is occupied by a metallic core, typically modeled as pure iron or a binary iron alloy, whereas the mantle and crust are often represented as layers with a mineralogy consistent with that of the Earth (Duffy et al. 2015). These so-called super-Earths are rocky planets with masses up to ten times that of Earth and radii up to 1.6 times Earth’s radius. In addition, super-Mercuries, which are characterized by relatively large iron-rich cores, are also studied (Adibekyan et al. 2021). In some cases, models are extended to include an additional outer layer of water/ice, as in so-called super-Ganymedes, or water incorporated in the mantle (e.g., Sotin et al. 2007). Other models consider mantles composed of exotic materials, such as silicon carbide (Nisr et al. 2017; Miozzi et al. 2018).
2. **Temperature and pressure dependence of transport and material properties**
  The thermal and structural evolution of planetary interiors is strongly influenced by the properties of their constituent minerals, including key transport properties such as (1) viscosity and (2) thermal diffusivity, as well as thermal expansion. These properties are mostly dependent on interatomic distances, which can be approximated by density (i.e., Birch’s law). Density, in turn, is highly sensitive to variations in pressure and temperature. An extensive review of the pressure and temperature dependence of these properties is provided by Si (2014). In summary, density (ρ) depends primarily on pressure (P), with temperature having only a small effect. It can be expressed as:
  5 $$ \frac{\rho}{\rho _{0}} = \left (1 + \frac{K_{0}'}{K_{0}} P \right )^{ \frac{1}{K_{0}'}}, $$
  where $\rho _{0}$ is the density at zero pressure, $K_{0}$ is the bulk modulus at zero pressure, $K_{0}'$ is the pressure derivative of the bulk modulus at zero pressure, i.e., $K_{0}' = \left ( \frac{dK}{dP} \right )_{P=0}$. Thermal and transport properties are challenging to constrain experimentally. Therefore, formulations have been proposed by various authors to derive them from routinely measured physical quantities (e.g., density and thermal expansion). Here, we present as an example the empirical relationship between viscosity and melting temperature used to estimate the dependence of viscosity on pressure and temperature, following Si (2014) (see Table 1).

**Table 1:** Density (and temperature) dependence of key physical properties. $H_{0}^{*}$ is the reference activation enthalpy

Property	Density (and Temperature) Dependence
Thermal expansion	$\dfrac{\alpha}{\alpha _{0}} = \left ( \dfrac{\rho _{0}}{\rho} \right )^{K_{0}'}$
Grüneisen parameter	$\dfrac{\gamma}{\gamma _{0}} = \left ( \dfrac{\rho _{0}}{\rho} \right )$
Thermal diffusivity	$\dfrac{\kappa}{\kappa _{0}} = \left ( \dfrac{\rho _{0}}{\rho} \right )^{2 + \frac{3}{2}K_{0}' - \frac{11}{6}} \left ( \dfrac{T_{0}}{T} \right )$
Melting temperature	$\dfrac{T_{m}}{T_{m0}} = \exp \left [ 2\gamma _{0} \left (1 - \dfrac{\rho _{0}}{\rho} \right ) + \dfrac{2}{3} \log \left ( \dfrac{\rho _{0}}{\rho} \right ) \right ]$
Viscosity	$\dfrac{\eta}{\eta _{0}} = \exp \left [ \dfrac{H_{0}^{*}}{R T_{m0}} \left ( \dfrac{T_{m}(\rho )}{T} - \dfrac{T_{m0}(\rho _{0})}{T_{0}} \right ) \right ]$
3. **Volatiles in the core and mantle and their effect on rheology**
  The concentrations of volatiles (such as hydrogen, carbon, and sulfur) in terrestrial planets are inherited from the conditions under which proto-planets formed and are challenging to constrain (Peslier et al. 2017). Temperature plays the primary role in controlling the composition of condensed matter in the proto-planetary nebula and, in turn, depends on the distance from the host star. Close to the star, high temperatures inhibit the condensation of volatiles, resulting in volatile-poor materials (e.g., Broadley et al. 2022). Farther from the star, where temperatures are lower, volatiles can condense. The boundary separating these regions is known as the “snow line” (Hayashi 1981). Because most rocky planets form inside the snow line, they are generally expected to contain relatively small amounts of water and other volatiles.
  A significant fraction of the water found on terrestrial planets likely originates from the mixing of materials that formed beyond the snow line. In addition, nebular ingassing has also been proposed as a possible mechanism for delivering water to planetary interiors (Sharp 2017). Once incorporated into the building blocks of a planet and not lost through early outgassing, water can dissolve into the newly forming planet (Horn et al. 2025). Its abundance is controlled by the chemistry and physical properties of the interior, as well as by the processes that operate during differentiation and evolution (Thompson 1992; Williams and Hemley 2001). During the early stages of planet formation, the outer regions of a planet consist of completely molten silicate, forming a global magma ocean, while an iron-rich core segregates through iron–silicate differentiation. At this stage, substantial amounts of water (in the form of hydrogen) can dissolve into the interior, as both the molten mantle and the core have the potential for high storage capacity (e.g., Hirschmann et al. 2012; Li et al. 2020; Tagawa et al. 2021; Dorn and Lichtenberg 2021; Miozzi et al. 2025). However, the extent to which water is incorporated into the core depends on the specifics of the core–mantle separation process, which is largely controlled by the depth of the magma ocean (Si 2023). As the planets cool and minerals begin to crystallize, the water storage capacity of the interior decreases because the solubility of water in crystallized phases is lower than in melts and outgassing becomes very efficient (e.g., Bolfan-Casanova 2005; Guimond et al. 2023) and (Guimond et al. 2026, this collection) and (Steinmeyer et al. 2026, this collection).
  Water plays a crucial role in shaping planetary interiors by influencing the chemical and transport properties of main mantle components, with important consequences for mantle’s chemistry and structure (e.g., Green et al. 2010). In particular, the presence of water reduces mantle viscosity and promotes melting. During partial melting, water preferentially partitions into the melt, leaving the residual solid relatively dry.
  In rocky planets, mantle convection can induce partial melting in localized regions. Once generated, melt separates from the solid residue and migrates upward or downward depending on its density relative to the surrounding material. When water-rich melts ascend toward the surface, water is released into the atmosphere and oceans. The amount of outgassed water depends on the partial pressure of water in the atmosphere, with higher atmospheric water pressures reducing outgassing efficiency (Steinmeyer et al. 2026, this collection). On planets with active plate tectonics or surface mobilisation, however, surface water can be recycled back into the mantle through subduction processes (e.g., Hirschmann 2006). During subduction, water is transported into the deep interior within hydrated oceanic crust and serpentinized mantle minerals, which progressively release water as pressure and temperature increase.

#### Crust Properties

The crust on rocky planets is derived from partial melting of the underlying mantle. For the silicate rocks typical of Earth and solar system planets, partial melting produces a magma richer in silica with less magnesium and iron when compared to the mantle. This magma is buoyant relative to the mantle (at least at shallow upper mantle pressures) and will therefore rise to the surface. Upon solidification, a distinct, more silicic rock is formed at the planet’s surface, composing the crust.

On Earth, there are two main types of crust with different compositions due to their distinct formation processes. The oceanic crust that covers the seafloor is predominantly made of basalt, which results from partial melting of the mantle and solidification of the ensuing magma. The continental crust is more silica rich, making it less dense than the oceanic crust. This lower density, combined with the fact that continental crust is typically thicker than oceanic crust, causes the continents to stand at a higher elevation than the oceanic crust. As a result they are mostly exposed subaerially, with the oceans filling in the basins formed by the lower elevation oceanic crust.

The crust can exert a significant influence on the dynamics of the mantle and the tectonics of rocky planets. Because of its distinct composition compared to the mantle, crust is positively buoyant. This is in fact by definition true for the crust regardless of the planet type or composition; crust can only exist if it is less dense than the underlying mantle. Otherwise, magma would not have risen to the surface to form a crust in the first place, or this crust would later have been lost into the mantle by gravitational instability. The positive buoyancy of the crust due to its distinct composition acts against negative thermal buoyancy that drives downwelling of cold surface material through mantle convection, either in the form of subduction for a plate-tectonic planet, or drips from the base of the lithosphere on a stagnant-lid planet.

The thickness of the oceanic crust then exerts a strong control on whether an oceanic plate can subduct. A thicker oceanic crust would require an even thicker lithospheric mantle beneath the crust to achieve negative buoyancy (Korenaga 2006). As oceanic crust thickness increases with increasing mantle temperature due to more extensive melting, at least assuming formation at mid-ocean ridges like in modern Earth, hotter mantle temperatures may impede subduction by preventing plates from reaching net negative buoyancy (Davies 1992; van Thienen et al. 2004b). However, basalt will transition to eclogite, a rock denser than the mantle, at $\sim 50\text{ km}$ depth on Earth, so convective thickening of crust can trigger sporadic subduction as a result (van Thienen et al. 2004a). This process may be enhanced on larger planets, where higher gravity causes the eclogite transition to occur at shallower depths. Furthermore, melting also creates a layer of depleted mantle that experienced partial melting. This depleted layer is highly viscous and becomes part of the lithosphere. A thicker crust will then also lead to a thicker lithosphere, such that net negative buoyancy of plates may still be achieved (Korenaga 2006), at least for an Earth-like composition.

The crust’s buoyancy as controlled by its thickness and composition is then critical to whether a planet can operate in a plate-tectonic regime. If plates cannot become net negatively buoyant, subduction, and therefore plate tectonics, cannot operate. Planets with different bulk compositions could produce different magmas and, hence crusts, from partial melting of the mantle (Brugman et al. 2021). Planet compositions prone to producing particularly thick or buoyant crusts would then be less likely to exhibit plate tectonics (Unterborn et al. 2017). However, crust production on planets with non-Earth-like compositions is just beginning to be explored, so there are still large uncertainties over what kinds of crusts would be produced in the end. Furthermore, additional study of the dynamics of mantle convection on planets with thick, buoyant crusts is needed to fully assess when such crusts prevent subduction and plate tectonics.

The crust with its different thermal properties, such as reduced thermal conductivity and enrichment in radioactive elements relative to the mantle, is also important for the planet’s thermal evolution (see Sect. 3.3), as seen in our solar system. Crusts on Solar System rocky planets are typically basaltic, as they form from primary partial melting of the mantle, similar to the Earth’s oceanic crust. However, the lack of recycling at subduction zones allows this crust to build up and progressively thicken over time (e.g., Breuer and Moore 2015; Byrne 2020).

#### Surface Temperature

The tectonic regime of a rocky planet is controlled, among other factors, by the surface temperature, which also influences surface heat fluxes, and thereby the cooling rate and convective vigor of the mantle. One of the most important effects of surface temperature concerns the stability and availability of liquid water at the surface, which reduces the friction coefficient when it seeps into the deeper lithosphere through thermal cracking and hydration, and acts as a lubricant in subduction zones (Korenaga 2007). This lubrication is essential for sustaining plate tectonics over geological timescales. Consequently, even modest increases in surface temperature can have global, long-term consequences and can ultimately shut down plate tectonics altogether (Lenardic et al. 2008).

Beyond its effect on water stability, surface temperature can also directly influence the surface tectonic regime. Lower surface temperatures lead to a thicker and stronger lithosphere (Byrne et al. 2021a), which is more resistant to failure and favors the development of a stagnant-lid regime. At the other end, high surface temperatures can shift the lithosphere and crust from a brittle to a ductile rheological regime. In such cases, surface deformation and recycling may occur through lithospheric weakening due to magma intrusion (the plutonic-squishy-lid scenario; Lourenço et al. 2020) or through convective mobilization of a sluggish surface (Noack et al. 2012). Both scenarios may have played a role in the tectonic evolution of Venus. High surface temperatures may also enhance grain growth of minerals in the lithosphere, potentially stabilizing the surface of hot planets such as Venus by hindering the initiation of plate tectonics (Landuyt and Bercovici 2009). Research on the effect of surface temperature on tectonic regimes has become increasingly relevant in the context of tidally locked exoplanets, where strong day–night temperature contrasts may lead to different tectonic regimes on opposing hemispheres (Van Summeren et al. 2011; Meier et al. 2021).

Finally, surface temperature also influences the thermal evolution of the interior of a planet both indirectly and directly. Indirectly, it affects the surface tectonic regime, and regimes characterized by efficient surface mobility, heat-pipe volcanism, or plutonic intrusion experience increased mantle cooling efficiency. Directly, surface temperature controls the temperature profile across the crust and lithosphere, thus regulating the heat flux from the mantle. This, in turn, influences lithospheric thickness and the overall energy budget of the mantle (see also Sect. 3).

#### Intrusion/Extrusion Ratio

Another factor influencing the active tectonic regime of a planet, and therefore its thermal and compositional state, is the ratio of intrusive to extrusive magmatism. This ratio reflects the proportion of melt generated above the depth of neutral buoyancy that is emplaced as surface volcanics versus intrusive plutons within or at the base of the crust (Vogt et al. 2012). Its controlling mechanisms are not fully understood, but likely involve a combination of factors such as lithospheric thickness, crustal density, structure and stress state, magma generation rate, volatile content, and planetary size (i.e. gravity). Many of these evolve over a planet’s history, altering the intrusion to extrusion balance and, in turn, affecting and being affected by the active tectonic regime. On Earth, intrusion efficiencies are generally estimated at 80–90% (extrusion efficiencies of 10–20%), depending on tectonic setting (Crisp 1984). The highest extrusion efficiency reported by Crisp (1984) is about 60%. Low extrusion efficiency appears to be a persistent feature throughout Earth’s evolution, with values near 20% providing a better match to early Earth observations (Rozel et al. 2017). On Venus, it is also thought that intrusion efficiency is high because Venus’ lithosphere is expected to be warm and soft due to high surface temperature, which favors plutonism (Gerya 2014). Notably, magmas within Mercury are likely lower density than those of Earth and the other inner Solar System worlds because of lower FeO abundances in the planet’s bulk silicate portion (Vander Kaaden and McCubbin 2015). As a result, the intrusive:extrusive ratio for Mercury could plausibly be closer to unity than for any other known terrestrial body (Byrne et al. 2018).

The intrusion–extrusion ratio influences the active tectonic regime and, consequently, the mechanisms of recycling and planetary heat loss. For planets lacking large-scale subduction, two end-member cases can be identified: the heat-pipe regime (100% extrusive) and the plutonic–squishy-lid regime (>80% intrusive). In the heat-pipe regime (Sect. 2.2.5), heat is efficiently transported via melt migrating through lithospheric fissures (“heat pipes”). However, over time, sustained volcanic activity thickens the lithosphere, which insulates the mantle and reduces cooling efficiency, unless other sources of heating act, such as tidal heating (Lourenço et al. 2018). In contrast, high intrusion efficiency promotes a transition to the plutonic–squishy-lid regime (Sect. 2.2.4). This occurs across a wide range of surface yield stresses and even in models without plastic yielding (Lourenço et al. 2020). Here, hot magma intrusions weaken the lithosphere, leading to drips, delaminations, and motion of blocks or small plates at the surface. Intrusive magmatism acts to thin the lithosphere, leading to sustained recycling of overlying crustal material and more effective mantle cooling than in volcanism-dominated planets (Lourenço et al. 2018). The plutonic–squishy-lid regime may be relevant to Venus. Observations that Venus’ lowland plains are fragmented and moving through time (Byrne et al. 2021b), and evidence for lithospheric thickness and heat flow comparable to Earth (Smrekar et al. 2023), are more consistent with this regime, at least in the lowlands, than with episodic- or stagnant-lid tectonics. Upcoming Venus missions may provide data to further test this hypothesis.

### How Likely Is It for a Planet to Experience Plate Tectonics?

As the number of known rocky exoplanets continues to grow, understanding which tectonic regimes they experience (and may have experienced in the past) becomes central to understanding their long-term evolution. The tectonic regime influences interior cooling, atmospheric build-up and recycling, magnetic field generation, and therefore habitability and the potential for life. Plate tectonics is widely regarded as a key ingredient in sustaining Earth’s habitability over geological time and in supporting the conditions under which life evolved (see Sect. 5). However, observationally, Earth remains the only known inhabited world. If plate tectonics is indeed important for life, a fundamental question follows: how likely is it for a rocky planet to experience plate tectonics?

Rocky planets are expected to convect because accretion, differentiation, and long-lived internal heating (radiogenic, and in some cases tidal) typically leads to mantle Rayleigh numbers far above the critical threshold for convection (Sect. 2.1). In this sense, the key question is not whether a planet convects, but what the surface expression of mantle convection is (i.e., tectonic regime) and whether it can sustain long-lived, global recycling via subduction. Plate tectonics is therefore one outcome within a spectrum of tectonic regimes (mobile lid, episodic lid, stagnant lid, heat pipe, plutonic-squishy lid, etc), and a planet may transition among these regimes through time or even experience different regimes at the same time at different locations. Importantly, identical parameters can lead to multiple stable tectonic states (hysteresis), implying that a planet’s evolutionary path and early events (e.g., impacts, early melting, and water loss) can be as decisive as its present-day size or heat budget.

The potential of a planet for having plate tectonics depends on the competition between processes that can be expressed using two nondimensional ratios: (1) the ratio of yield stress to lithospheric buoyancy, and (2) the ratio of yield stress to viscous stresses. The ratio of these two quantities is equivalent to the Rayleigh number, as discussed in Coltice (2023). Lithospheric failure is highly sensitive to deformation mechanisms, making volatile content and surface temperature critical. Water lowers viscosity, promotes melting, and, critically, reduces effective friction, and strengthens lubrication, especially in subduction zones, thus allowing sustained subduction on Earth. Higher surface temperatures can dry the surface of a planet and shut down this lubrication, while also driving grain growth that may strengthen hot lithospheres. On the other end, low surface temperatures thicken and strengthen the lid, favoring stagnant-lid behavior. Thus, Earth-like surface conditions are not uniquely required for mantle convection, but they may be ideal for maintaining a relatively weak lithosphere that supports continuous subduction at present. However, it is important to remember that even on the early Earth, elevated mantle temperatures may have inhibited the operation of modern-day plate tectonics (van Hunen and van den Berg 2008; Fischer and Gerya 2016). Finally, the rheology of the uppermost mantle plays a critical role in favoring specific tectonic regimes, as it controls the degree of coupling between the lithosphere and the underlying convecting mantle. On Earth, the lithosphere moves atop a low-viscosity asthenosphere, which may result from variations in water content in the sublithospheric mantle (Si 2012), the presence of distributed partial melt (Hua et al. 2023), dislocation creep (Arnould et al. 2023; Patočka et al. 2024), and/or the accumulation of hot material from mantle plumes beneath the lithosphere (Morgan et al. 2013). This layer is believed to stabilize plate tectonics by decoupling small-scale mantle convection, associated with Earth’s relatively high Rayleigh number, from the large-scale motion of tectonic plates (Lenardic et al. 2006; Coltice et al. 2019). Although its absence on other planets does not necessarily preclude surface mobility, it may favor a more episodic tectonic regime (Tackley 2000b).

Crustal production further complicates this balance. Crust is intrinsically buoyant relative to its mantle source, and thick or especially buoyant crust counteracts the negative thermal buoyancy of cooling plates and can prevent net negative buoyancy, inhibiting subduction. This effect is offset by mechanisms such as basalt to eclogite phase change at depth, or the development of a depleted lithospheric mantle due to efficient melting. Because melting and crust composition depend on bulk planet composition, the likelihood of plate tectonics may vary widely across rocky planets, simply because there is no sufficient driving force for plate tectonics (e.g., Kite et al. 2009; Unterborn et al. 2017).

Internal structure and heating history also play a role by controlling convective stresses and cooling efficiency. Thin mantles (large core-to-mantle ratios) cool more rapidly, while radiogenic or tidal heating can sustain vigorous convection, although enhanced heating may favor alternative tectonic regimes (e.g., heat-pipe or plutonic–squishy-lid tectonics) rather than classical plate tectonics.

Overall, plate tectonics likely occur for a relatively narrow parameter space where (1) plates can fail (often aided by water and moderate surface temperatures), (2) the lithosphere can become negatively buoyant despite the stabilizing effect of buoyant crust, (3) convective forcing and thermal evolution do not steer the planet toward other tectonic regimes, and (4) the mantle is sufficiently cool to transmit stresses to the lithosphere, as higher mantle temperatures tend to favor stagnant-lid behavior or lithospheric drippings. Because many of these controls depend on history and strong feedback mechanisms (volatile cycling, crustal growth, exogenic processes such as impacts), the occurrence of plate tectonics may be rare and not universal.

## Thermal and Chemical Evolution of Rocky Planets

### Setting the Initial Stage: Initial Temperature Distribution and Mantle Composition After Magma Ocean Solidification

The long-term evolution of a rocky planet is usually considered after the crystallization of the magma ocean, i.e., after the onset of solid-state convection in the mantle. If a magma ocean crystallizes rapidly, in less than a few million years (e.g., Solomatov 2015), the temperature profile in the crystallized layer follows the crystallization temperature (typically set to the solidus temperature), while the solid mantle below follows the accretion temperature (Breuer and Moore 2015). If the solidification of the magma ocean proceeds more slowly (over timescales of ∼10–100 Ma), for example due to a dense and thermally insulating atmosphere (Lebrun et al. 2013), or due to the formation of an insulating floating crust as on the Moon, the solidifying mantle likely convects before it is completely crystallized (but the solid particles are already interconnected in a solid matrix) (Maurice et al. 2020). This process is enhanced for fractional crystallization (separation of melt from solidifying mantle crystals) as the progressively iron-enriched solid becomes denser with time. Due to the typical crystallization from bottom to top in a magma ocean, melt fractionation leads to an unstable configuration in which density increases toward the surface. This can trigger a convection process in the mantle, resulting in a temperature profile that follows the adiabatic curve in almost the entire mantle immediately after the magma ocean solidifies. Depending on the assumption of the core formation process, the core may be in thermal equilibrium with the mantle or initially superheated relative to it. In the latter case, a thermal boundary layer forms at the core-mantle boundary, with important implications for the early evolution and sustainability of planetary magnetic fields (see Sect. 4). The surface temperature, in turn, is controlled primarily by stellar irradiation and atmospheric composition (Steinmeyer et al. 2026, this collection).

After the crystallization of the magma ocean, mantles are often assumed to be compositionally homogeneous, but this assumption is only valid under certain conditions, namely if the magma ocean crystallizes in equilibrium and no fractionation occurs between melt and crystallized material. Such conditions may be approximately met during very rapid solidification. However, even the formation of a primary crust implies that this assumption is not correct, since extraction of crustal material necessarily leaves behind a depleted mantle. Thermal models of Earth’s magma ocean suggest that crystallization initially proceeds close to equilibrium, with significant melt–solid separation occurring only during the final stages of solidification, when crystallization rates decrease (Solomatov 2015). Fractional crystallization can therefore lead to a compositionally layered mantle, as commonly assumed for the lunar mantle. Although the resulting unstable density stratification can drive rapid mantle overturn and promote efficient mixing, complete homogenization is unlikely, as indicated by the existence of early-formed mantle reservoirs in the Earth revealed by isotopic signatures (Williams et al. 2021). There is also geochemical evidence that the mantle of Mercury is compositionally heterogeneous (Weider et al. 2015).

Another possibility for early mantle layering and thus an inhomogeneous mantle is the formation of a basal magma ocean (BMO) at the core mantle boundary, produced by the accumulation of dense, iron-rich silicate melt enriched in heat-producing elements (Labrosse et al. 2007). A detailed discussion of the various ways in which a BMO can form on Earth can be found in Labrosse et al. (2015), Boukaré et al. (2025) and for Mars in Samuel et al. (2021). A BMO within Venus has also been suggested (O’Rourke 2020). The presence of a BMO has important implications for a planet’s thermochemical evolution, particularly by reducing the efficiency of core cooling and thereby influencing the evolution of the magnetic field - although a dynamo might have also operated within the BMO itself (Ziegler and Stegman 2013; Blanc et al. 2020; O’Rourke 2020; Schaeffer et al. 2025; Dragulet and Stixrude 2025). Similar questions regarding the formation, long-term evolution, and magnetic consequences of BMOs have also been explored for large super-Earths (Soubiran and Militzer 2018; Luo and Deng 2025; Lherm et al. 2026).

### Long-Term Solid State Convection

**Fig. 2:**
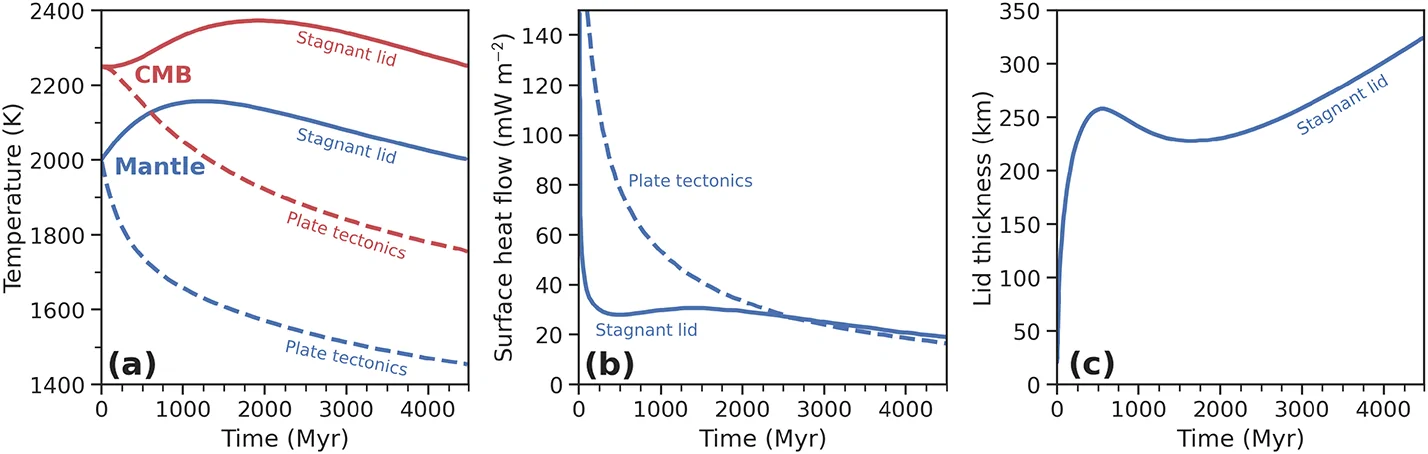
Representative evolutions of mantle temperature (a, blue lines), core-mantle boundary (CMB) temperature (a, red lines), surface heat flow (b), and stagnant-lid thickness (c) for stagnant-lid convection (solid lines) and plate tectonics (dashed lines). Note that 4500 Myr corresponds to the present day. After Breuer and Moore (2015)

After the solidification of the magma ocean, the long-term evolution of a planet’s interior is driven by secular cooling, which is the gradual, slow loss of the internal heat to space. The cooling rate and internal temperature distribution within the planet depend on the tectonic regime. For example, plate tectonics promotes efficient cooling by transporting cool surface material deep into the mantle. In contrast, a stagnant-lid regime thermally isolates the mantle from the surface, resulting in higher mantle temperatures and inefficient cooling (Fig. 2). Importantly, melting and crust production can have important effects on the thermal (and chemical) evolution of a planet (e.g., Lourenço et al. 2016), potentially giving rise to plutonic–squishy lid or heat-pipe regimes.

#### Modeling the Mantle Evolution with Parametrized Convection

Numerical modeling has been a fundamental tool for investigating mantle dynamics and long-term thermal evolution. Although state-of-the-art 2D or 3D models provide the most detailed representation of mantle convection, they are computationally expensive and time-consuming. In many applications, the spatially resolved convection patterns produced by these models are less important than the evolution of bulk mantle properties, such as average mantle temperature or crustal thickness.

Parameterized convection models offer a simpler and computationally efficient alternative. These 1D models compute the globally averaged mantle temperature based on the energy balance between heat lost through the planet’s surface and heat production from radiogenic decay, heat input from core cooling, and other energy sources (see Sect. 2.1.1). Rather than explicitly resolving convective flow, mantle convection is parameterized using scaling laws derived from laboratory experiments and 2D and 3D numerical convection models, which describe global radial heat transport (e.g., Thiriet et al. 2019).

For a convecting mantle with an adiabatic temperature profile, boundary layer theory (BLT) is used to calculate the thermal boundary layers at the top and bottom of the mantle, through which heat is transported by conduction. The basis of every parametrized convection model is an energy equation for each reservoir. A minimal set of equations for the mantle and core takes the form:

6 $$\begin{aligned} \rho _{m} c_{m} V_{m} \frac{\mathrm{d}T_{m}}{\mathrm{d}t} &= -q_{m} A_{m} + q_{c} A_{c} + Q_{m} V_{m}, \end{aligned}$$

7 $$\begin{aligned} \rho _{c} c_{c} V_{c} \frac{\mathrm{d}T_{c}}{\mathrm{d}t} &= -q_{c} A_{c} . \end{aligned}$$

where $T _{\mathrm{m}}$ and $T _{\mathrm{c}}$ are the temperature of the mantle and core, ρ, c, V, A are density, specific heat capacity, volume and area of the mantle (subscript m) and core (subscript c), $q _{\mathrm{m}}$ is the heat flux across the top boundary layer of the mantle, $q _{\mathrm{c}}$ is the heat flux across the core-mantle boundary, and $Q _{\mathrm{m}}$ is the volumetric radiogenic heat production rate (e.g., Spohn 1991).

For a vigorously convecting mantle, the fluxes $q _{\mathrm{m}}$ and $q _{\mathrm{c}}$ can be obtained from boundary layer theory by determining the thickness of the top and bottom thermal boundary layers. The thickness of the thermal boundary layers scales with the Rayleigh number (see Eq. (1)) of the convecting mantle as:

8 $$ \delta _{\mathrm{u}} = D _{\mathrm{m}} \left ( \frac{Ra}{Ra _{\mathrm{crit}}}\right )^{-\beta}, $$

where $D _{\mathrm{m}}$ is the thickness of the convecting mantle and β is the Nusselt-Rayleigh parameter. This parameter describes how the thickness of the upper boundary layer scales with the Rayleigh number and is calibrated using laboratory experiments and 2D/3D numerical simulations. A commonly used value for β is 1/3; however, some studies suggest β<1/3 and that this exponent also depends on the tectonic regime. The tectonic regime is typically prescribed rather than determined self-consistently. For a planet operating in the plate tectonic regime, the energy balance equations given in Eqs. (6) and (7) are applied directly. In contrast, for a planet in the stagnant-lid regime, the mantle is commonly divided into a stagnant lid and an underlying convecting mantle. This formulation requires an additional energy equation at the lid–mantle interface, as well as solving a heat conduction equation in the lid to model its time evolution. This approach also allows the incorporation of melting, melt transport, and crust formation, which requires further modifications to Eqs. (6) and (7) (e.g., Morschhauser et al. 2011).

An alternative approach to BLT is mixing-length theory (MLT). MLT estimates local energy transport by considering the distance a fluid parcel can travel before dissipating its thermal energy. Thus, it provides a way to self-consistently calculate the temperature at every point throughout the mantle. In contrast, BLT provides only limited depth-dependent information because it assumes an adiabatic mantle. MLT is therefore particularly well suited to model planets with partially molten mantles (e.g., Bower et al. 2018).

One-dimensional parameterized models necessarily capture only the global average thermal state. Due to their much lower computational cost, parametrized convection models allow for the exploration of far wider parameter spaces, making them particularly well suited for exoplanet modeling, where large uncertainties in planetary parameters are common.

Figure 2 shows results from one-dimensional parameterized mantle convection models illustrating the thermal evolution of a rocky planet operating in a plate tectonic regime compared to one in a stagnant-lid regime. In the plate tectonic case, both the CMB temperature and the average mantle temperature decrease more rapidly due to the higher surface heat flux, particularly during the first ∼2.5 Gyr. In contrast, surface heat loss in the stagnant-lid regime is much less efficient, leading to the progressive growth of a thick, insulating lithospheric lid over time.

### Crust Formation

Planetary crusts are commonly divided into three types: primary, secondary, and tertiary (e.g., Taylor 1989; McLennan 2022; Cawood et al. 2025). Primary crusts form through the crystallization of a surface magma ocean during the late stages of accretion. The feldspathic lunar highland crust is the most prominent example, with another candidate possibly being a graphitic flotation crust on Mercury. Secondary crusts, which are typically basaltic in composition, form later through pressure-release partial melting of solid planetary mantles. Examples include the crusts of Mars and Venus, part of Mercury’s crust, Earth’s oceanic crust, and the lunar maria (Byrne 2020). Tertiary crusts are formed by partial melting of reworked secondary (basaltic) crust. Earth’s continental crust is the only confirmed example of a tertiary crust, although some of Venus’ tessera regions have been proposed as a possible additional example (e.g., Gilmore et al. 2023). Water, mantle rock, and eroded continental crust play key roles in its formation process. In the following, we discuss the formation mechanisms of the different crust types and how they influence the tectonic regimes and the thermochemical evolution of rocky planets.

#### Primary, Secondary, and Tertiary Crust

Primary crusts are formed through the crystallization of a magma ocean. The first minerals to crystallize are olivine and pyroxene, which sink as a result of their high density. As solidification progresses, the remaining melt becomes increasingly enriched in iron and other incompatible elements, such as radioactive elements, making the crystallized material near the surface denser than the material below. This configuration is dynamically unstable, and this dense crust formed from the crystallization of the magma ocean preferentially sinks into the mantle. As a result, primary crusts are expected to be transient and are unlikely to be preserved (except possibly in small quantities) on the surfaces of Earth, Venus, or Mars. The situation is different for smaller bodies such as the Moon. Due to lower pressures, plagioclase, which is lighter than the melt, crystallizes after approximately 80% of the magma ocean has solidified. It rises and forms the feldspathic lunar highland crust, which today makes up most of the moon’s crust today. An unstable stratification still develops within the mantle, similar to that described above, but occurs below the primary crust. In the case of Mercury, data from the MESSENGER mission suggests that it was formed under highly reducing conditions, meaning there is little free oxygen. Under such conditions, carbon can exist in its elemental form as graphite, which is buoyant relative to silicate melt. This may have allowed a primary graphite crust to form (Keppler and Golabek 2019). A thick primary graphite crust has also been predicted for rocky exoplanets forming in protoplanetary disks around stars with photospheric C/O ratios greater than 0.65, or in other systems through radial transport of refractory carbon (Moriarty et al. 2014).

Secondary crusts form through partial melting of upwelling material in the convecting mantle, when adiabatically decompressing mantle rocks cross the solidus. Partial melting generates buoyant magmas that either intrude into the near-surface lithosphere or erupt onto the surface, where they crystallize to form crustal rocks. On Earth, the oceanic crust constitutes a relatively thin layer, typically less than ten kilometers thick. Most of this crust is composed of mid-ocean ridge basalt (MORB), which forms at relatively low pressures beneath spreading centers. Intraplate ocean-island basalts contribute a smaller fraction of the oceanic crust and originate from deeper mantle sources. Large basaltic plateaus, such as the Ontong Java Plateau in the western Pacific, further add to the compositional and structural complexity of the oceanic crust (Taylor and McLennan 2003). Within the framework of plate tectonics, the oceanic lithosphere can be viewed as a conveyor belt that transports newly formed crust, covered by a thin layer of sediments derived from biological activity in the oceans and erosion of continental crust, from mid-ocean ridges to subduction zones (e.g., Taylor and McLennan 2003). This recycling process carries volatiles from the surface into the planet’s interior, playing a crucial role in regulating planetary climate and maintaining surface conditions conducive to life (e.g., Kasting and Catling 2003; Cockell et al. 2016), as well as in the generation of tertiary crust. On the modern Earth, subduction is driven by the negative buoyancy of oceanic plates as they sink into the mantle. However, because oceanic crust is positively buoyant, plates do not attain net negative buoyancy until the underlying cold, dense lithospheric mantle has thickened sufficiently to overcome the positive buoyancy of the crust (Oxburgh and Parmentier 1977); on the modern Earth this occurs when plates are older than ∼30 Myrs old (Afonso et al. 2007).

Despite the broadly basaltic nature of secondary crusts, their compositions exhibit significant variability, likely reflecting differences in formation processes and subsequent evolutionary histories. Although all planetary crusts are depleted in volatile elements relative to chondritic compositions, they differ markedly in their abundances of incompatible elements, in SiO_2_ content and in total alkali concentrations (Na_2_O + K_2_O). For example, Mercury’s crust is comparatively felsic, approaching the composition of Earth’s continental crust, while the Martian crust is more mafic, similar to Earth’s oceanic crust, and the Venusian crust appears to be more mafic still. These comparisons should be understood as generalizations about average crustal composition, since individual planetary surfaces are likely to display substantial local and regional lithologic diversity, as can be observed on Earth.

In the context of modern plate tectonics, subduction zones are the sites where tertiary continental crust is formed by partial melting of subducted oceanic crust, sediments, and mantles rocks in the presence of subducted water (which lowers the solidus temperature) (e.g., Campbell and Taylor 1983; Stein and Ben-Avraham 2015). Continental crust does not subduct due to its buoyancy and, despite its greater longevity compared to oceanic crust (having a median surface age of approximately 600 Myr (Goodwin 1996) versus about 65 Myr for oceanic crust), is continuously modified by weathering and erosion. The resulting sediments are transported to subduction zones by surface water flow. In addition, continental crust is eroded by friction between the continental keel and the subducting slab (e.g., Stern 2011). Subduction and crust production rates are closely linked to mantle flow through the plate subduction rate, which is, in turn, governed by the rheological properties of mantle rocks.

While it is widely accepted that some form of plate tectonics operated throughout the Proterozoic (since 2.5 Ga), the mechanisms by which proto-continents formed during the Archean remain debated (e.g., Cawood et al. 2013, 2022; Chowdhury et al. 2025; Rey et al. 2024). Proposed mechanisms include underplating, as well as the thickening and cratonization of volcanic plateaus through partial melting of mafic crust and crustal reworking. The energy driving these processes is generally thought to derive primarily from mantle heat and volcanic activity. Recycling of crust/lithospheric mantle might still also have been possible, perhaps when a squishy lid is present, leading to drips and crustal delamination. However, it should be noted that the rock record becomes increasingly sparse with age. Moreover, the age distribution of continental crust rock can be explained by models assuming some form of continuous continental crust production and recycling (including plate tectonics) occurring throughout the Archean (e.g., Dhuime et al. 2012; Korenaga 2021a; Höning and Spohn 2023).

Observational constraints on the area of continental crust are derived from the age distribution of continental rocks (Goodwin 1996), variations in freeboard through time, and geological and geochemical evidence (e.g., Cawood et al. 2022; Chowdhury et al. 2025). These constraints indicate that approximately 50% of the present-day continental crust is younger than 1.3 Gyr, whereas only about 25% is older than 2 Gyr. Models that link crustal production rates to the vigor of mantle convection - as plate tectonics models do (e.g., Höning and Spohn 2023) - and the recycling rate to the area of the continental crust predict growth rates that decrease over time as the mantle cools, alongside recycling rates that increase as continents expand. Consequently, rapid early crust formation is expected, potentially evolving toward a quasi-equilibrium state characterized by a roughly constant continental surface area.

Would Earth-sized, or even Earth-like, rocky exoplanets necessarily develop a similar ratio of continental to oceanic crust surfaces, and thus similar land/water surface ratios? This ratio may be of astrobiological significance, as emergent land surfaces provide direct access to solar irradiation and serve as major sources of nutrients through weathering, erosion, and the transport of material to the oceans (e.g., Glaser et al. 2020; Lingam and Loeb 2019; Höning and Spohn 2023) (Guimond et al. 2026, this collection). Recent studies have argued that this is not necessarily the case (e.g., Höning et al. 2014; Höning and Spohn 2016, 2023). They propose that the present continental configuration of the Earth maximizes the total length of subduction zones that intersect continental margins, therefore enhancing crustal production, sediment subduction, and slab-related basal crustal erosion. In contrast, stable end-member configurations would either maximize or minimize continental coverage, minimizing subduction-zone length, and leading to either a predominantly land-covered planet or an ocean world. While the former may be a desert planet lacking water, the latter may be nutrient-poor. The present Earth state would be marginally stable, perhaps kinetically stabilized by the decreasing mantle temperature, but would maximize continental crust-related geodynamic activity and disequilibrium.

#### Influence on Thermal and Dynamic Evolution

The generation of melt, its transport to the surface, and the associated crust formation play a key role in the planetary heat engine, contributing to more efficient cooling. When mantle material melts, latent energy is consumed and initially removed from the system:

9 $$ \frac{dE_{lat}}{dt} = L \rho _{m} \frac{dV_{m}}{dt}, $$

with $E_{lat}$, being the latent energy, L the latent heat, $\rho _{m}$ the melt density, and $V_{m}$ the volume of melt. As the buoyant melt rises, it crystallizes as extrusions on the surface or as intrusions within the crust or upper mantle. The latent heat is then released as the melt cools rapidly to the ambient temperature (e.g., the surface temperature in the case of extrusion). In this way, heat is efficiently transported from the melting region toward the surface in the form of latent heat, and mantle temperatures are strongly buffered by this process at the solidus temperature. Mantle melting also redistributes radiogenic heat sources. Because radioactive elements behave incompatibly, they become concentrated in the melt and are transported toward the surface, leading to a crust that is enriched in heat-producing elements relative to the mantle. On Earth, the oceanic crust is enriched by a factor of 5 and the continental crust by a factor of 20. On Mars, recent data from the InSight mission indicate that the basaltic crust is enriched by a factor of 10–15 (Knapmeyer-Endrun et al. 2021). This means that the mantle is depleted of radioactive heat sources by about 60% and its heating is reduced.

The presence of a crust can also substantially slow planetary cooling because crustal materials generally have lower thermal conductivity than mantle rocks. Part of this contrast arises from compositional differences, as minerals typical of the crust conduct heat less efficiently than those of the mantle. Under standard conditions, compact basalt has a thermal conductivity of ∼2–2.5 W/(m⋅K) and compact mantle rock ∼3–5 W/(m⋅K) (note that thermal conductivity generally decreases with temperature and increases with pressure). However, material structure, particularly porosity, may have a much stronger influence on thermal conductivity than composition alone (e.g., Horai 1976). This effect is especially pronounced for bodies with thin or negligible atmospheres, where pore spaces are effectively vacuum-filled. Because vacuum has a lower thermal conductivity than atmospheric gasses, heat transport through the pores is inefficient. On airless bodies such as Mercury and the Moon, where impact processes have produced heavily fragmented crustal material, near-surface thermal conductivities can be as low as ∼0.01–0.001 W/(m⋅K). At depths of a few meters to tens of meters, the thermal conductivity can increase to ∼0.1–1 W/(m⋅K), depending on the degree of compaction and the presence of larger rock fragments. In general, the thermal conductivity increases with depth as the material becomes more consolidated and less porous. For larger bodies, the compaction is more efficient and the so-called outer regolith or mega-regolith layer is thinner. Furthermore, pores filled with water or ice can also increase the effective conductivity (although this enhancement may be reduced in the presence of saline brines or impurity-rich ice). The latter is assumed to be the case on Mars, where an effective crustal conductivity of ∼2 W/(m⋅K) is assumed. On the Moon, the assumed value is 1.5 W/(m⋅K) or less. A fundamentally different situation arises if a thick graphite crust is present. Graphite has a thermal conductivity one to two orders of magnitude higher than those of silicate rocks (∼20–200 W/(m⋅K)). Under such conditions, conduction is the dominant mode of heat transport.

Whether cooling of the planetary interior or thermal blanketing by the crust dominates depends on the crustal production rate and the structure of the crust, and this balance may evolve over time. During the early evolution of a planet following the magma ocean phase, when mantle temperatures are close to melting conditions, cooling associated with secondary magmatism can therefore exceed heat transport by solid-state convection. Under conditions of intense melting and crust formation, a planet lacking active plate tectonics may operate in either a heat-pipe or a plutonic–squishy lid tectonic regime, depending on whether most melt is erupted at the surface (heat pipe) or retained predominantly as intrusions within the crust and lithosphere (plutonic–squishy lid; see descriptions above in 2.2). If melt production rates are sufficiently high, both regimes can cool the interior efficiently (Lourenço et al. 2018). The distinction between these regimes and plate tectonics is more evident in the resulting temperature structure: plate tectonics leads to cooling of the whole interior, while heat-pipe and plutonic–squishy lid regimes primarily cool the upper mantle where melting occurs, leaving the lower mantle comparatively warmer. Despite this similarity, the heat-pipe and plutonic-squishy lid regimes differ significantly. In the heat-pipe regime, efficient melt transport to the surface, coupled with the downward advection of cold material, leads to rapid growth of a thick and cold stagnant lid. In contrast, in the plutonic-squishy-lid regime, repeated hot intrusions warm the crust and lithosphere, maintaining a relatively thin lid.

Later in a planet’s evolution, as the interior cools and magmatic activity decreases, the insulating effect of the crust becomes increasingly important due to its reduced thermal conductivity and the redistribution of heat-producing elements. A strongly insulating crust may also facilitate long-term crust production in smaller, faster-cooling bodies. This effect can be seen on the Moon, for example, where young basaltic units with ages of ∼2 Gyr are observed (Li et al. 2021; Che et al. 2021). Such prolonged magmatism is unlikely for planets with predominantly graphite crusts. These planets cool more rapidly than those with silicate crusts and are therefore expected to generate a lower proportion of secondary crust (Hakim et al. 2019).

Beyond its role in planetary cooling, the crust also influences internal dynamics and the spatial distribution of volcanism. Two- and three-dimensional convection models indicate that mantle upwellings, and thus magmatic activity, preferentially develop beneath regions of enhanced crustal thickness. An example is Mars, which exhibits long-lived and spatially stable volcanism over several billion years in the Tharsis and Elysium regions (Plesa et al. 2018). The longevity and distribution of magmatic activity may also play an important role in planetary habitability, as magma can supply important nutrients and energy sources for life and contribute to atmospheric replenishment through volcanic outgassing.

### Influence of Tectonic Regime on the Redistribution of Basalt/Eclogite

**Fig. 3:**
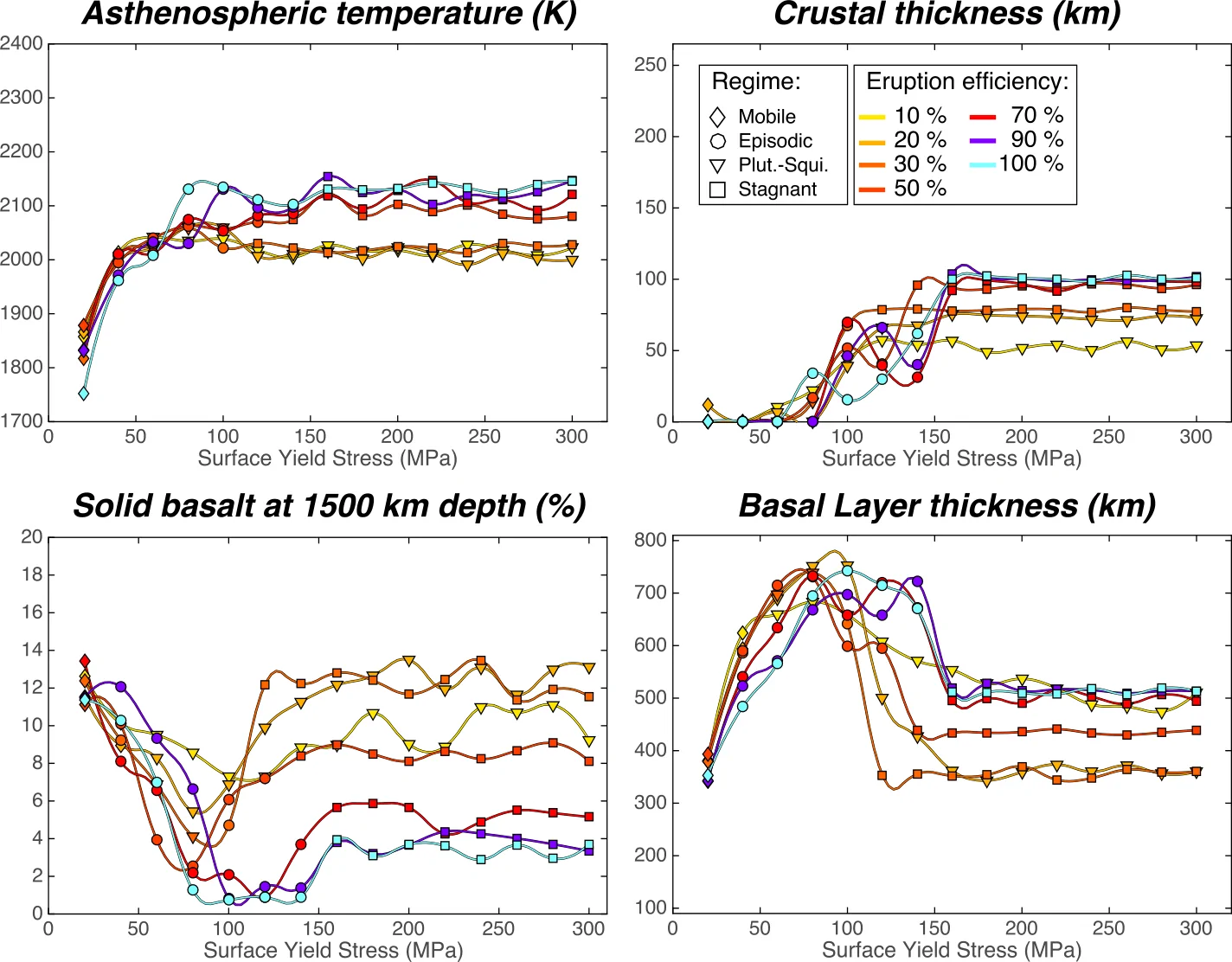
Asthenospheric temperature (top left), crustal thickness (top right), internal composition represented by the solid basalt fraction at 1500 km depth (bottom left), and basal layer thickness (bottom right) averaged over the last 500 Myr for mantle convection simulations run with the code StagYY (Tackley 2008) for 4.5 Gyr. The surface yield stress is the specified threshold surface stress in numerical models above which the material response shifts from viscous flow to brittle deformation (i.e., breaks). The eruption efficiency is the proportion of melt generated above the depth of neutral buoyancy that is erupted (the rest is intruded). Different eruption efficiencies are represented by curves of different colours linking different symbols, which represent different tectonic regimes. Figure adapted from Lourenço et al. (2020); see that study for further details on the simulations

Figure 3 shows an overview of mantle temperature and how basaltic material is distributed in the mantle by the end of the numerical runs simulating different tectonic regimes for an Earth-size planet. Mantle temperatures can only be understood by looking at the compositional state of the mantle after billions of years of convection and recycling. Here, we look at four quantities from the work of Lourenço et al. (2020) aiming to show the importance of the tectonic regime to the internal state of a rocky planet and its heat engine over time: (1) internal temperature, in Fig. 3 (top left) computed as the interior temperature averaged over the 200 km below the point of minimum viscosity in the upper mantle, (2) basaltic crust thickness, in Fig. 3 (top right), (3) mid-mantle-depth basalt fraction, in Fig. 3 (bottom left), and (4) basal layer thickness in Fig. 3 (bottom right), which represents the thermochemical piles formed near the core-mantle boundary (CMB) following subduction or any other process that brings lithospheric basaltic material into the deep mantle. Initially, the basalt fraction is 20% everywhere. All the values in Fig. 3 are averaged over the last 500 Myr of evolution of the planet.

#### Stagnant Lid

In cases with a stagnant lid, crustal thicknesses are large and strongly depend on extrusion efficiency: the higher the extrusion efficiency, the thicker becomes the crust. Crustal thicknesses of up to a hundred kilometers can be obtained (Fig. 3, top right). Such amounts of crust can only be stored because of the absence of both subduction and intrusive magmatism (which weakens the lithosphere). Cases with low eruption efficiency have thinner crusts because plutonism eases eclogitic delaminations by warming up the crust. In contrast, a higher eruption efficiency leads to a strong lithosphere and allows for the buildup of a thick crust.

A thick crust/lithosphere insulates the mantle from the surface, leading to higher mantle temperatures (Fig. 3, top left) and to a strongly depleted mantle (Fig. 3, bottom left) through volcanic (heat) piping. As a result, the mantle cannot melt and cool efficiently. Depletion is most pronounced in the upper mantle, but also extends into the middle and lower mantle.

Mantle viscosity further influences internal structure. Higher reference viscosities increase the thickness of thermal boundary layers in convecting fluids (e.g., Fowler 1985), which in turn favors the formation of thicker crust (see example in Fig. 2). A thicker crust reduces the efficiency of conductive cooling at the surface of the planet, leading to higher internal temperatures. In addition, higher reference viscosities suppress the recycling of eclogite from the bottom of the lithosphere into the deep mantle, resulting in thinner basal layers (Fig. 3, bottom right).

#### Episodic Lid

In the episodic-lid regime, the alternation of overturns and stagnant phases leads to midmantle basalt contents that are strongly variable, but amongst the lowest for the various tectonic regimes. The basaltic crust is rebuilt during the stagnant phases (starting during the overturn itself) and may resurface again over time. Therefore, crustal thickness values strongly vary depending on the number of overturn events. In general, crustal thicknesses increase with increasing yield stress, as the number of overturns decreases. Furthermore, the closer in time to the present that an overturn event last happened, the thinner will be the crust as there is no time for a thick crust to form (again). Successive resurfacing events result in thick basal layers around the CMB (Armann and Tackley 2012; Lourenço et al. 2016). The basal layer thickness strongly increases with increasing yield stress, up to 800 km. Regarding energy loss, even if the number of overturns in the evolution of a planet is generally low, a lot of heat is released during them. This, combined with a thick basal layer insulating the core, leads to lower internal temperatures compared to planets covered with a stagnant lid (Fig. 3, top left).

#### Mobile Lid

Small crustal thicknesses are obtained for the cases in a mobile-lid regime, due to the efficient recycling of the lithosphere caused by continuous subduction, and lower internal temperatures due to efficient heat loss. The basalt content of the midmantle is always relatively high, indicating that basalt is efficiently (re)cycled into the lower mantle. This efficient mixing in the mantle leads to thin basal layers compared to other tectonic regimes.

#### Plutonic-Squishy Lid

In the plutonic-squishy-lid regime, due to high intrusion efficiencies, the lithosphere remains too warm to grow. Due to the thin lithosphere in this regime, conductive heat flow becomes large and mantle temperatures are lower than that of a stagnant lid. Crustal thicknesses increase with increasing yield stress until a relatively high constant value is reached. Hot eclogite constantly drips into the convecting mantle from the bottom of the crust. Part of it is entrained in the mantle circulation keeping the internal ambient mantle enriched, whereas the other part settles and forms a thick basal layer at the CMB. Basal layer thicknesses are high, similarly to what occurs in the episodic-lid regime. These large basal layer thicknesses show that eclogite drips coming from the bottom of the lithosphere are not only restirred into the mantle but are also efficiently deposited near the CMB. A thick basal layer and a large surface heat flow are responsible for the significantly low internal temperatures.

### Influence of Planetary Mass

With the discovery of the first rocky exoplanets, which, due to observational biases, were initially found to be more massive than the rocky bodies of the Solar System, numerous geodynamic studies began to investigate how planetary mass influences interior heating, volcanic activity, plate tectonics, magnetic field generation, atmospheric evolution, and habitability.

From a first-principles perspective, an increase in planetary mass is expected to lead to higher interior temperatures when assuming similar heat sources (e.g., comparable accretional energy per unit mass and constant radiogenic heat production per unit mass). As planetary mass increases and assuming the same composition, planetary radius R also increases. Given that internal heat production scales with volume ($\propto R^{3}$) and heat loss through the surface scales with surface area ($\propto R^{2}$), the more massive the planet, the more heat it can retain. Increasing heat for more massive planets should then trigger stronger volcanism (Kite et al. 2009), although the amount of melt that is buoyant enough to reach the surface may be limited by increasing planetary mass, both due to higher pressures that raise mantle melting temperatures with depth and due to the reduced buoyancy of the melt at high pressures (Noack et al. 2017).

Another consideration is whether a metal-enriched basal magma ocean, as proposed for the early Earth (Labrosse et al. 2007), could form in more massive rocky planets. The much higher melting temperatures expected at the core-mantle boundary of such planets would make the formation of a basal magma ocean more difficult, unless the mantle did not cool efficiently after the formation of the planet (for example, due to a whole mantle overturn).

The timing and longevity of secondary melt production further depend on assumptions regarding the post-magma-ocean thermal structure. If a whole-mantle overturn occurs immediately after magma ocean solidification (Elkins-Tanton 2008), the mantle may cool significantly and generate large amounts of secondary melt before long-term secondary outgassing begins. Under stagnant-lid conditions, the onset of secondary volcanism on more massive planets may therefore be delayed, although once initiated it may persist for longer (Dorn et al. 2018). Volcanic activity is also closely linked to the surface tectonic regime. On Earth, plate tectonics accounts for almost all of the present-day volcanism. However, for massive rocky planets, cooling associated with subduction may preferentially affect the upper mantle, potentially reducing melt production there, while leaving the lower mantle relatively insulated (Stamenkovic et al. 2012), depending on the adopted rheological model (Tackley et al. 2013).

Regarding the initiation of plate tectonics, higher mantle temperatures are generally expected to lead to faster mantle convection. This relationship can either make plate tectonics more efficient by increasing convective stresses (Valencia et al. 2007) or suppress it by enforcing a decoupling of the strongly convecting mantle and the lithosphere by creating a higher viscosity contrast between mantle and surface (O’Neill et al. 2007). These opposing effects have led to contrasting predictions concerning the likelihood of plate tectonics on super-Earths. Numerous subsequent studies have explored this topic by examining the combined effects of heat production, rheology, and mantle Rayleigh number. These results, summarized in Ballmer and Noack (2021), suggest that the occurrence of plate tectonics on super-Earths is a multi-parameter problem rather than a simple function of planetary mass alone.

## Magnetic Field

The generation of a magnetic field is strongly linked to the planetary heat engine, in which the kinetic energy of fluid motion is converted into magnetic field energy. The question of the existence of a magnetic field is particularly interesting, as it is hypothesized that maintaining a habitable surface (i.e., with stable surface water) over long periods of time may require a magnetic field to protect the atmosphere from mass loss and retain large amounts of water, as well as to protect life on the surface from radiation (Dehant et al. 2007; Lammer et al. 2008). However, the general assumption that a magnetic field protects the atmosphere has changed somewhat in recent years due to missions such as Cluster (2001–present) observing Earth magnetosphere, Mars Express (2004–present), the Mars Atmosphere and Volatile EvolutioN (MAVEN) mission (2014–2025), and Venus Express (2006–2014). Comparisons between the planets of the solar system show that an intrinsic magnetic dipole field is not required to prevent the escape of planetary atmospheres caused by the stellar wind, and that the presence of such a field can instead increase the rate of ion escape (Ramstad and Barabash 2021). Even if this matter has not yet been conclusively clarified, the presence of a self-generated magnetic field and also of an earlier magnetic field, which is determined for the planets in our solar system by measuring remanent magnetization in the crust, can provide important information about the thermal evolution of a planet in general.

### How to Generate a Magnetic Field

The generation of a self-sustained magnetic field within a rocky body requires convection within the electrically conducting metallic core and at least slow rotation. Rotation helps to align the flowing molten metal, enabling it to self-organize through sustained electric currents. This is necessary for maintaining a global magnetic field. However, the minimum rotational speed required is very low and is commonly found present in rocky planets. Typically, a distinction is made between thermally, chemically, or mechanically driven dynamos. The latter are rather exotic, with mechanical stirring of the core caused through precession and the associated differential motion (Dwyer et al. 2011) or by changes in the rotation rate due to large impacts (Le Bars et al. 2011). The following section outlines the conditions under which the most common dynamos, namely thermal and chemical dynamos, occur.

**Thermal Dynamo** Thermal convection in the fluid core, as in the mantle, is driven by a sufficiently large superadiabatic temperature difference between the core and the mantle. It occurs if the core is chemically homogeneous and the heat flux, $q_{c}$, exceeds the heat flux conducted along the core adiabat, $q_{ad}$, which therefore provides a criterion for the existence of thermally driven core convection:

10 $$ q_{c} > q_{ad} = k_{c} \left | \frac{dT}{dr} \right |_{ad} = \frac{k_{c} \alpha _{c} g_{c} T}{C_{p}} $$

where $k_{c}$ is the thermal conductivity of the core, $\left | dT/dr \right |_{ad}$ is the adiabatic temperature gradient in the core, $\alpha _{c}$ is the thermal expansivity of the core, $C_{p}$ is the core heat capacity at constant pressure, and $g_{c}$ and T are the gravitational acceleration and temperature at the core-mantle boundary (CMB). The range of the critical heat flux in the literature varies widely. For instance, it is in the range of 5 and $20~\mathrm{mW}\,\mathrm{m}^{-2}$ for Mars (Nimmo and Stevenson 2000) and $25\mathrm{--}100~\mathrm{mW}\,\mathrm{m}^{-2}$ for the Earth (Konôpková et al. 2016; Pozzo et al. 2012). The large variation is mainly because the thermal conductivity of iron-rich material under high pressures and temperatures and depending on the composition, i.e. the amount of light elements, is not well known and also controversially discussed (Ohta and Hirose 2021). Direct measurements of solid iron using a dynamically laser heated diamond-anvil cell place the thermal conductivity at Earth’s core-mantle boundary near the low end with values of $18\mathrm{--}44~\mathrm{W}\,\mathrm{m}^{-1}\,\mathrm{K}^{-1}$ (Konôpková et al. 2016; Hsieh et al. 2020). Ab initio calculations of transport properties suggest that the thermal conductivity at the CMB is much higher with values of 90 to $150~\mathrm{W}\,\mathrm{m}^{-1}\,\mathrm{K}^{-1}$ (De Koker et al. 2012; Pozzo et al. 2012). High values also result from measurements of the electrical conductivity and applying the Wiedemann–Franz law to calculate the thermal conductivity (Gomi et al. 2013). It is important to note that the evolution of the magnetic field can vary greatly depending on the critical heat flow.

Regardless of the conflicting estimates of the critical heat flow, the core cooling is strongly controlled by the thermal evolution of the mantle above. If the mantle removes heat from the core at a rate that exceeds the critical heat flow, then the core will convect. In contrast, if the mantle removes heat at a rate less than the critical heat flow, the core is thermally stably stratified, and dynamo action by thermal convection is not possible.

**Chemical Dynamo** Unlike a thermal dynamo, a chemical dynamo is driven by compositional density variations. Typical processes that lead to a (local) change in composition include crystallization and mass transfer into and out of the fluid core. For the latter, examples include the introduction of iron-rich material with a composition different from that of the core material (e.g., Trinh et al. 2026) and the exsolution of lighter lithophile elements (rock-loving), such as Mg, from the core (e.g., Badro et al. 2016). Exsolution leaves behind a denser liquid, generating natural convection. The most significant factor contributing to compositional convection in the core, however, is the crystallization of iron-rich fluids with non-eutectic compositions. All terrestrial planets are generally assumed to contain light elements alloyed with iron in their cores (Sohl and Schubert 2015).

Since a variety of light elements such as S, Si, O, C, and H are conceivable in planetary cores, attention has been given to investigating, both experimentally and theoretically, the high pressure and temperature behavior for multi-component iron alloys. Phase diagrams correlating pressure-temperature and composition offer a first glimpse into the crystallization temperature and nature of the first phase to crystallize in planetary cores (Hirose et al. 2021). The relative densities of crystals and melt of the specific chemical systems then define the crystallization scenario. At present, Fe-FeS represents the most studied system, and will be used in the following paragraphs to describe crystallization scenarios that might be of relevance for terrestrial bodies.

#### Classical Earth-Like Dynamo

In the classical Earth dynamo, the crystallization process starts in the center of the core (i.e., the center of the planet) because the gradient of the core liquidus, $T_{cm}$, is larger than that of the core temperature, $T_{c}$ (thus $dT_{cm}/dP > dT_{c}/dP$). The composition lies on the iron-rich side of the eutectic, and iron crystals form first during cooling. Due to their high density, these crystals sink and form an inner solid core. As the inner core grows, the buoyant material at the inner core boundary rises and drives convection in the outer liquid core. The release of latent heat through crystallization at the boundary further strengthens the convection in the outer core.

The buoyancy release associated with this compositional change can even exceed the work done against stabilizing thermal stratification. Thus, in contrast to a purely thermal dynamo, a chemical dynamo also works when the heat flux out of the core is lower than the heat flux along the core adiabat. However, there is a critical value of the magnetic Reynolds number $R_{m}=(uD)/\lambda $ (a measure of flow velocity u in terms of shell thickness D and magnetic diffusivity λ) of about 50, below which a magnetic field will not be generated (Christensen and Aubert 2006). The dynamo would stop, although the inner core may still grow slowly over time. Nevertheless, it is generally expected that the classical Earth-like dynamo will be sustained for a long time, on the order of billions of years. The field can no longer be generated when a eutectic composition is reached (unless there is sufficiently strong thermal convection) or when the core is fully crystallized.

#### Iron-Snow and Other Alternative Crystallization Scenarios

The crystallization process can differ significantly from the scenario described above in smaller planetary bodies (even in bodies the size of Mars) because the pressure in the core is lower than in Earth’s core. Experiments on Fe-FeS show that the melting temperature profile can have a negative slope or a melting temperature profile that is flatter than the core temperature profile with $dT_{cm}/dP < dT_{c}/dP$, so that Fe first crystallizes at the core mantle boundary upon cooling. Because solid iron particles are heavier than the surrounding Fe-FeS liquid, they can fall as the so-called iron snow. A snow zone forms, in which the core temperature profile is equal to the melting temperature profile and which is limited from below at a depth where the core temperature is higher than the liquidus and the iron remelts.

As cooling progresses, the snow zone grows at the expense of the deeper, well-mixed liquid core. Finally, an inner core begins to grow when the snow zone encompasses the entire core. It is assumed that the dynamo is located in the iron-rich layer below the snow zone (Christensen and Wicht 2007). This dynamo would only be active in the period between the formation of the snow zone and the time when it reaches the center of the core and is shorter than the classical Earth-like dynamo described above.

This so-called iron-snow regime has been suggested to operate in the present-day cores of Ganymede (Rückriemen et al. 2015), Mercury (Wicht and Heyner 2014) and Triton (Tilke et al. 2026), and may also occur in the Martian core in the future (Stewart et al. 2007). Furthermore, Gaidos et al. (2010) proposed this crystallization mechanism for massive rocky exoplanets with masses of more than 2.5 Earth masses. Recent studies using high-energy lasers and in situ X-ray diffraction have measured the melting point of iron at up to 1000 GPa, which is three times the central pressure of the Earth’s core. These measurements suggest, however, that, as in the case of the Earth, the crystallization of the core occurs from the bottom up (Kraus et al. 2022).

Alternative crystallization scenarios are possible with a higher composition of sulfur. If the sulfur content of the core is greater than the eutectic concentration, solid FeS (or Fe_3_S_2_ for pressures between 14 GPa and 20 GPa, and Fe_3_S for even higher pressures) will precipitate within the core. It has been suggested that, with the exception of the scenario in which Fe_3_S crystallizes at the center of the core, all other scenarios may in principle produce a dynamo (Breuer et al. 2015). Crystallization, and thus the dynamo it produces, can last for a very long time, on the order of billion years, as is the case on Earth with the crystallization of iron. More recently, precipitation and transport of light elements, such as Si, O, and Mg, from the outer core into the lowermost mantle have been proposed as an additional mechanism that can trigger chemical convection (Badro et al. 2016; Hirose et al. 2017), although this process is unlikely to provide sufficient power to sustain a dynamo (Du et al. 2017, 2019).

### Magnetic Field Evolution

**Fig. 4:**
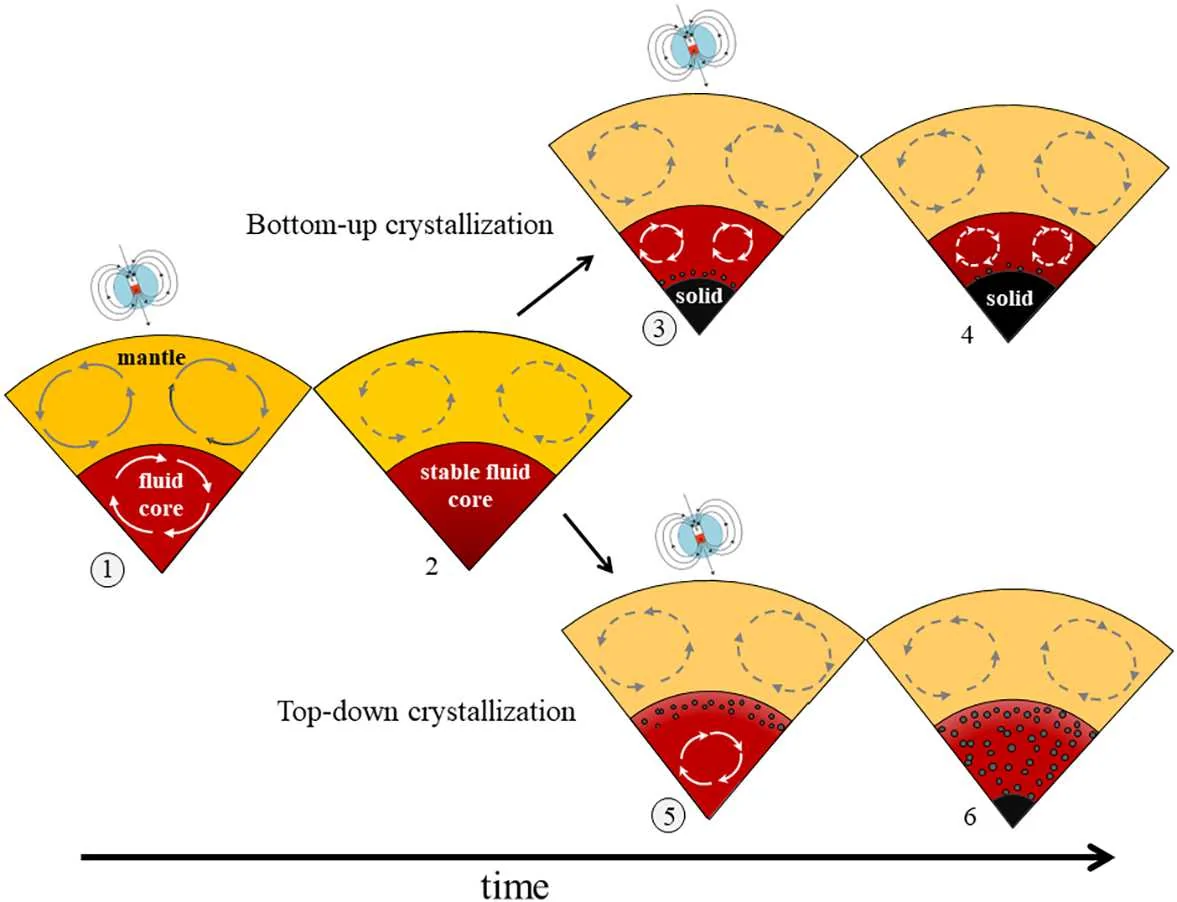
Possible scenarios of core evolution starting with a thermal dynamo. Time is increasing from left to right. The upper row is representative for bottom-up (Earth-case) freezing and the lower row for top-down freezing (iron snow). Solid lines with arrows indicate strong convection and cooling. Dashed lines with arrows indicate weak convection and cooling. Dotted lines with arrows in the core indicate sluggish convection not sufficient to overcome ohmic dissipation (no dynamo generation). Grey circles mark sinking iron crystals. The numbers 1–6 denote the different states of a cooling planet which may change in time and transit from one to another. Possible scenarios paths of the evolution are 1–4 or 1,2,5,6. Depending on the core composition there is no step 2 and the scenario goes from 1,3,4 or 1,5,6

Whether and when a terrestrial body can have a thermally or chemically driven magnetic field depends not only on the composition and properties of the core, but is ultimately determined by the thermal evolution of the mantle, which dictates the cooling rate of the core (e.g., Stevenson et al. 1983; Breuer et al. 2010). A typical scenario of thermal and magnetic field evolution is as follows: In the early evolution of a planet, i.e. after accretion and core formation, a thermal dynamo is active (Fig. 4(1)). At this early hot stage, the core is completely liquid and the cooling of the core is particularly effective, especially if the core is superheated compared to the mantle. This superheating is postulated for rapid core formation, which is assumed for the terrestrial planets in our solar system based on the measured Hf-W isotope compositions. How long the thermal dynamo then remains active and when the cooling of the core falls below the critical heat flow depends on various factors, including the tectonic regime and the associated effectiveness of cooling. Plate tectonics, which strongly cools the deep mantle and core (see above), can maintain a planet’s thermal dynamo for longer than stagnant lid convection, for which the interior cools more slowly. In the latter case, the core heat flux thus sinks below the critical heat flux more quickly (Fig. 4(2)). If the core cools below the melting temperature during further evolution, core crystallization takes place, and a chemical dynamo can be generated (Fig. 4(3) and (5)). Cores with a small amount of light elements and a high melting temperature start to freeze out earlier than a core with many light elements and low melting temperatures. It is important to note that a present-day thermal dynamo is unlikely for all terrestrial planets and moons, as shown by thermal evolution models. Late and present-day magnetic fields in terrestrial bodies are typically associated with crystallization and therefore with a chemical dynamo. These relations demonstrate that terrestrial bodies are always somewhat on the borderline in terms of their ability to host a dynamo. For this reason, the individual evolution of a planet is highly significant. Simple criteria, such as the planet’s size, cannot be taken as a basis for this.

Mars is possibly a classic example of an early thermal dynamo and a present-day dynamically stable core that is still completely liquid (Fig. 4(2)), meaning that the present-day Martian interior is not cooling effectively enough for a thermal dynamo and the crystallization of the core has not yet begun (e.g., Breuer 2019). The fact that crystallization has not yet started is due to the combination of stagnant lid convection and the associated slow cooling, and the high concentration of light elements in the core (e.g., Greenwood et al. 2021). This evolution is consistent with the observed early magnetization of the Martian crust up to 3.7 Ga and the current lack of a magnetic field, but also with the recent InSight data on the size and density of the core (Stähler et al. 2021). However, the latest interpretation of seismic data from InSight suggests that Mars may indeed have an inner solid core measuring 600 km in size (Bi et al. 2025) (Fig. 4(4)) and possibly a basal magma ocean (Khan et al. 2023; Samuel et al. 2023), in which an early dynamo may also have originated as suggested also for other planets (e.g., Lherm et al. 2024). If correct, this would significantly change our understanding of the thermal and magnetic field evolution of Mars.

The thermal evolution and the observed evolution of Mercury’s magnetic field are examples of a planet with an early thermal dynamo and the later onset of a chemical dynamo (e.g., Breuer 2019). Today’s magnetic field is driven by the crystallization of the core (Fig. 4(3)). The onset of crystallization depends on the composition of the core and the melting temperature, but it is likely that there was a phase between the thermal dynamo and the chemical dynamo without an internally generated magnetic field (Fig. 4(2)).

The evolution of Earth’s magnetic field can be explained by an early thermally driven dynamo followed by a later chemically driven dynamo. Evidence for a geomagnetic field is preserved in magnetized crustal rocks dating back to approximately 3.7 billion years ago and continuing to the present day. There is no direct evidence for an earlier magnetic field, as little crust older than this age has been preserved due to continuous recycling by plate tectonics. Models of thermal evolution show a thermal dynamo up to the beginning of inner core growth around 1.5–2 billion years ago (some dispute about this age exists) when a chemical dynamo sets in. The chemical dynamo is still active up to the present day due to the continuous core cooling and growth of the inner core. However, this scenario is only possible if the thermal conductivity of the core is in the lower range of estimates, i.e. 25–40 W/(m⋅K). The duration and existence of an early thermal dynamo is highly dependent on the thermal conductivity of the core (e.g., Landeau et al. 2022), which, as described above, is not well understood. If the conductivities are in the upper range, i.e. 80–150 W/(m⋅K), as estimated from ab initio and thermal resistivity measurements, then the inner core must have formed later, i.e. less than 1 Ga ago. The high thermal conductivities of the core also imply a high critical heat flow, and even efficient cooling by plate tectonics is not sufficient to maintain the heat flow from the core above this value for a long time. This also suggests that the Earth’s magnetic field may have been generated by means other than thermal convection prior to the growth of the inner core, for example by the exsolution of buoyant magnesium or silicate oxides at the core-mantle boundary (Badro et al. 2016; Hirose et al. 2017), or possibly a magnetic field generated within a basal magma ocean sitting at the core-mantle boundary (Ziegler and Stegman 2013; Blanc et al. 2020).

Venus currently lacks a global magnetic field, and there is no evidence of remanent magnetization in its crust. However, this does not imply that Venus never had a magnetic field earlier in its evolution; rather, it may reflect the limited observational data available. Several scenarios have been proposed to explain the evolution of Venus’ magnetic field. One model predicts an early thermal dynamo similar to that inferred for Mars (Stevenson et al. 1983). The present-day absence of a dynamo could result from a fully liquid core that is no longer convecting, consistent with the idea that Venus cools less efficiently than Earth due to the absence of plate tectonics (Nimmo 2002). Alternatively, if Venus experienced episodic crustal recycling events, the most recent occurring ∼0.3–1 billion years ago, enhanced mantle and core cooling during these episodes could have temporarily powered a thermal dynamo. It is also possible that Venus formed an inner core and thus once sustained a chemical dynamo. In that case, however, present-day cooling may be too inefficient and inner-core growth too slow to provide sufficient energy to maintain a dynamo (O’Rourke et al. 2018). A basal magma ocean has also been proposed for Venus, in which an earlier magnetic field could have been generated (O’Rourke 2020). Constraining these evolutionary scenarios will require future missions to determine whether and when a magnetic field existed. Although Venus’s high surface temperature reduces the likelihood that crustal magnetization preserves evidence of a past dynamo, such preservation cannot be ruled out (O’Rourke et al. 2018).

The existence of a magnetic field in rocky planets more massive than Earth depends strongly on how material properties evolve with increasing pressure. If the viscosity in the lower mantles of large exoplanets is assumed to be comparable to that of Earth (implying that the temperature-induced reduction in viscosity is approximately balanced by the pressure-induced increase) the likelihood of sustaining a magnetic field increases with planetary mass. This is because the heat flux extracted from the core, $q_{c}$, is predicted to increase more rapidly with mass than the conductive heat flux along the core adiabat, $q_{ad}$ (Blaske and O’Rourke 2021). This situation is different for a stronger pressure dependence of the viscosity (e.g., Stamenkovic et al. 2012; Tachinami et al. 2010; Zhang and Rogers 2022). In this case, the lower mantle convects more sluggishly, and the core cools less effectively and only starts to form an inner core later, if at all. It appears that beyond a critical planetary mass, the thermal dynamo lifetime decreases abruptly due to the pressure effect caused by the increase in mantle viscosity. This critical mass depends strongly on the initial core-mantle superheating, $\Delta{T_{CMB}}$, and thus on accretion and core formation processes, as well as on the pressure dependence of viscosity (Zhang and Rogers 2022). It is also not entirely clear yet if the cores of massive rocky planets would actually be fully molten at the end of the formation stage due to the increased pressures and melting temperatures in their interior (Boujibar et al. 2020; Bonati et al. 2021), depending amongst others on the amount of light elements partitioning into the core, a process which is not yet well understood.

### Magnetic Field Strength and Topology

The prediction of the strength of the planetary magnetic field may be useful to assess the habitability of exoplanets (e.g., Lammer et al. 2009; Tarduno et al. 2014; Kubyshkina et al. 2026). Scaling laws have been proposed to predict the magnetic field strength from observable planetary parameters. A critical review of the scaling laws and a comparison with numerical dynamo calculations have been provided by Christensen (2010). Accordingly, the most convincing scaling law for the magnetic field strength (in the dynamo region) was found to be independent of the planetary rotation rate but dependent on the energy flux in the dynamo region available to balance the ohmic dissipation:

11 $$ B^{2} = \mathit{const.} R_{c}^{4/3}q_{c}^{2/3} $$

Here, B is the magnetic induction, $R_{c}$ the core radius (for a terrestrial planet) and $q_{c}$ is the core heat flux. More generally, to account for chemically driven dynamos, $q_{c}$ may be replaced by the buoyancy flux in the core. The constant in the above equation takes values $0<\mathit{const.}<1$ depending on the mode of how the dynamo is driven, on the efficiency of the conversion of energy into magnetic field energy and on whether the field has a strong dipole component or is largely multipolar (see Christensen et al. 2009; Christensen 2010). In the latter case, the resulting magnetic field strength is only about half that associated with a strong dipolar field. Similarly, a thermally driven dynamo is expected to generate a magnetic field whose strength is only a fraction of that produced by a chemically driven dynamo. The topology of the field was found to depend on the strength of the inertial forces relative to the Coriolis force in dynamo experiments and thus on the rotation rate. This may be particularly relevant for tidally locked exoplanets for which the rotation rate is equal to the orbital frequency and much lower than, for example, the Earth’s rotation rate (e.g., McIntyre et al. 2019). However, these planets may still have substantial albeit multipolar magnetic fields that could protect the planet against radiation and the stellar wind if their core and interior evolution were similar to that of the Earth.

## Habitability and Bio-Geodynamics of Rocky Planets

### The Relationship Between Global Tectonics and the Evolution of Life

**Fig. 5:**
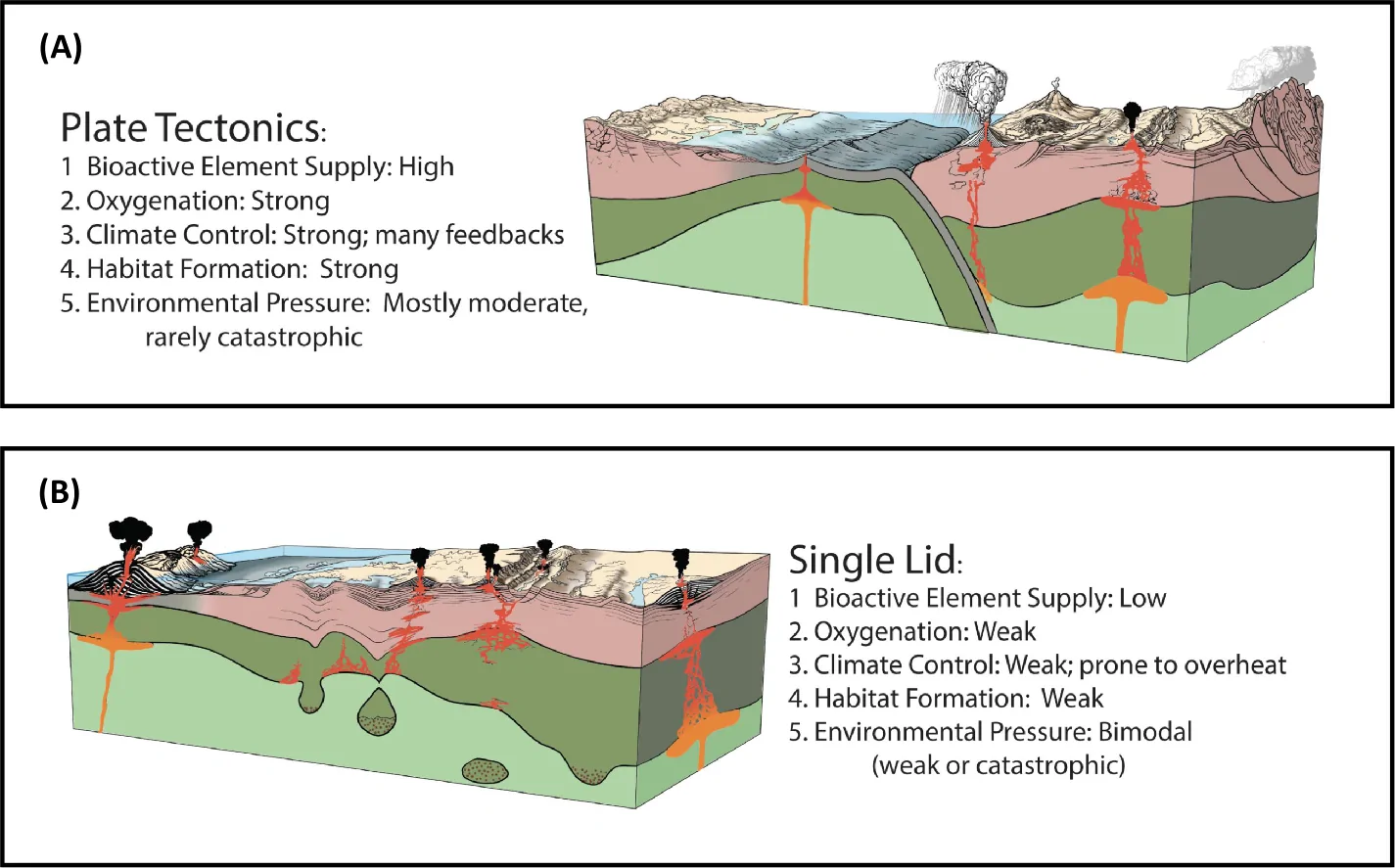
Summary diagram showing how plate tectonics can stimulate the emergence and evolution of life, whereas a single-lid tectonic regime may inhibit these processes. In this context, single-lid refers to a planetary tectonic style lacking plate tectonics and may include heat-pipe, plutonic–squishy-lid, or stagnant-lid regimes. Adapted from Stern and Gerya (2023)

The global tectonic style has repeatedly been suggested to play a key role in shaping habitability conditions and life evolution on rocky planets (e.g., Ward and Brownlee 2000; Stern 2016; Bédard 2020; Stern and Gerya 2024; Cawood et al. 2025). This is also a key subject of biogeodynamics, a transdisciplinary field that focuses on understanding and quantifying the interaction between the interior and surface evolution of Earth and Earth-like planets to modulate landscapes, atmospheres, oceans, climates, and life emergence and evolution (e.g., Zerkle 2018; Spencer 2022; Stern and Gerya 2023). Among the different possible tectonic styles of rocky planets (Fig. 1), plate tectonics is considered one of the key conditions for complex and intelligent life for the motives summarized in Fig. 5, though it may be rarely achieved (Ward and Brownlee 2000; Stern 2016; Bédard 2020; Stern and Gerya 2024). From this perspective, plate tectonics, characterized by a global mosaic of plates, has been contrasted with single-lid tectonics (in particular, heat-pipe, plutonic-squishy lid, and stagnant-lid tectonics, Fig. 5) to demonstrate their differences in biogeodynamic processes and nutrient cycles (Bédard 2020; Stern and Gerya 2023). Based on available geological data, five key biogeodynamical factors have been identified as probably controlling the accelerated development of complex life on Earth, shaped by modern-style plate tectonics (Fig. 5, Stern and Gerya (2023)): 1) increased nutrient supply; 2) increased oxygenation of the atmosphere and ocean; 3) long-term climate amelioration; 4) increased rate of habitat formation and destruction; and 5) moderate, sustained pressure from incessant environmental change. Most of these factors involve the presence of coexisting oceans and sub-aerial continents (that is, large areas of dry land), which is considered yet another (even more rarely achieved) global key condition for the emergence of life and accelerated evolution (e.g., Stern and Gerya 2024 and references therein, and Spohn et al. (2026, this collection), and Guimond et al. (2026, this collection)). Next, we review these five key biogeodynamical factors.

#### Nutrient Supply

The supply of nutrients is essential for life, especially key compounds containing bioactive elements such as organic carbon, ammonium, ferrous iron, and phosphorus (e.g., Zerkle 2018). Phosphorus is essential because it is a globally limiting nutrient that plays a unique role in marine biogeochemistry and ecology and consequently in evolution. In modern plate tectonics, in the presence of subaerial continents, phosphorus is primarily derived from the weathering of the continental crust and is delivered to the ocean by rivers (Föllmi 1996), suggesting strong climatic controls of its delivery through erosion and weathering. Tectonic and related orographic processes that expose fresh rock surfaces are crucial for enhancing the delivery of phosphorous and other inorganic nutrients, because soil shielding reduces nutrient fluxes due to chemical weathering (Hartmann et al. 2014). In this context, the microbial enhancement of carbon and sulfate acid weathering is considered an important driver of nutrient delivery to the oceans via rivers. This process also depends on the atmospheric oxygen level (Retallack 2022). Therefore, the addition of phosphorus, iron and other nutrients from erosion and weathering of collisional mountain belts created by plate tectonics is considered as one of the key processes that stimulates life and evolution.

#### Free Oxygen in Ocean and Atmosphere

During Earth’s geological evolution, free oxygen in the ocean and atmosphere increased nonmonotonously with time (see discussion in Steinmeyer et al. (2026, this collection)). This increase is explained by the proliferation of photosynthetic cyanobacteria (Fig. 5) combined with the efficient burial of organic carbon. This biogeodynamical process may have been further enhanced by the gradual oxygenation of the mantle through serpentinization of oceanic plates, which produces oxidized hydrated mantle rocks and molecular hydrogen. This process is combined with global subduction of the oxidized oceanic lithosphere, volcanic arc degassing, and the gradual loss of molecular hydrogen from the atmosphere (e.g., Duan et al. 2022; Zhu et al. 2026).

Another potential link between plate tectonics and atmospheric oxygenation has been suggested by Gaillard et al. (2011), who argue that the production of continental crust and exposed land allowed subaerial volcanism, as opposed to mostly submarine volcanism during the early evolution of the Earth with a flatter topography. This change in outgassed sulfur species from H_2_S to SO_2_ supported the oxygenation of the atmosphere.

#### Climate

Climate is especially important for complex life forms, such as plants and metazoans. Primitive life can exist between temperatures close to the freezing point of water and ∼120 °C, but complex life thrives between 5 °C and 35 °C. Biogeodynamically, plate tectonics and single-lid tectonics control climate in different ways. Plate tectonics and the related supercontinent cycle influence climate in four primary ways (Stern and Gerya 2023, 2024, and references therein). First, volcanic and plutonic degassing can warm or cool the climate, depending on the magma composition, which is controlled by the plate tectonic setting. CO_2_ emissions associated with mid-ocean ridge and mantle plume igneous activity enhence atmospheric warming. In contrast, explosive volcanism associated with convergent margins injects SO_2_ into the stratosphere, causing short-term cooling (Schmidt et al. 2012). Second, the proportion of the Earth’s surface covered by water versus subaerial land exerts a strong influence on the climate. The climate is more temperate when the proportion of water surface is high and harsher when there is more subaerial land. Due to continued long-term variations in sea level, plate tectonics is associated with periodicity in climate. Warmer (greenhouse) climates occur approximately 100 million years after continental breakup as new oceans widen (Worsley and Nance 1989). Less is known about the geodynamic processes controling sea-level variations during various single-lid tectonic styles, but they likely change less vigorously, more locally, and more discontinuously than during plate tectonics. Third, the weathering of silicate rocks consumes atmospheric CO_2_, so mountain building, which exposes more silicate rocks, results in atmospheric cooling (West et al. 2005). Enhanced erosion and weathering, which are associated with uplifts that accompany continental rifting and orogenesis, release more nutrients like phosphorus and iron that stimulate photosynthetic life. If sufficient dead organic carbon is buried, it sequesters CO_2_, which cools the climate. On a single-lid Earth-like planet, uplifts and erosion should be lower, so nutrient fluxes should be reduced, affecting the climate less. Fourth, subduction removes large volumes of marine carbonate rocks and organic carbon. This removes carbon and CO_2_ from the near-surface and sequesters them in the mantle, leading to climate cooling (Plank and Manning 2019).

It remains debatable what the carbon cycle and climate controls are for single lid planets (Foley and Smye 2018; Höning et al. 2019; Nakagawa and Iwamori 2019). The two active single-lid planets in our Solar System – Venus and Mars - have atmospheres that are >95% CO_2_ thereby suggesting a reduced efficiency of CO_2_ recycling on these planets compared to Earth. On the other hand, global-scale models show that the carbon cycle could potentially be efficiently maintained on such planets in particular with high volcanic extrusion rates (Foley and Smye 2018; Höning et al. 2019), where carbon recycling out of and into the mantle could occur through continuous volcanism, weathering, burial, sinking and delamination of carbonated crust. It has therefore been suggested that plate tectonics may not be required for establishing a long-term carbon cycle and maintaining a stable, habitable climate (Foley and Smye 2018).

#### Habitat Formation and Destruction

Habitat formation and destruction are integral parts of plate tectonics through the Wilson and supercontinent cycles, which govern landscape and climate evolution. Plate tectonics creates, fragments, and destroys habitats much faster and more efficiently than can active single-lid tectonic regimes can. The pace of evolution in relation to continental fragmentation and habitat formation has also been established (Valentine and Moores 1970; Zaffos et al. 2017; Liu et al. 2024).

#### Moderate Sustained Pressure on Organisms

Plate tectonics involves moderate sustained pressure on organisms from continuous environmental change, but this is probably not the case for single-lid regimes (Stern and Gerya 2023, 2024). Nutrient fluxes, topography, climate, and habitats continuously change over time with plate tectonics. Strong coupling of tectonics with erosion produces complex and variable landscape, climate, and precipitation patterns, which are especially pronounced along active plate margins. This complexity stimulates biodiversity (Pellissier et al. 2018). Continental rifting and plate divergence produce large continental shelves with robust sediment and nutrient delivery from adjacent continents. Currents and tides efficiently redistribute nutrients in shelves, creating favorable environments for marine life (Pellissier et al. 2018). In addition, mountainous continental rift escarpments can cause high endemic biodiversity on evolving subaerial continental margins, as discussed in Liu et al. (2024). These regions evolve through precipitation-driven erosion and the landward retreat of the high-relief topography. This creates transient habitat organization through mechanisms such as catchment expansion, the isolation of highland remnants, and the formation of topographic barriers. The resulting habitat isolation and reconnection on a time scale of millions of years serves as an allopatric speciation pump, where repeated geographic isolation and reconnection of populations promote the formation of new species, particularly creating the observed biodiversity of plants (Liu et al. 2024). All of these processes are stimulated by plate tectonics and the Wilson cycle, causing life to rapidly evolve and diversify. In contrast, single-lid tectonics is much less capable of exerting moderate, sustained environmental pressure. Changes here are likely controlled by the dominance of mantle plume-lithosphere interactions, which are generally more rapid and abrupt than plate tectonic processes (e.g., Burov and Gerya 2014; Gerya et al. 2015). This is especially apparent when mantle plumes form large igneous provinces (LIPs). Their most dramatic climatic effect is rapid global warming due to the increased release of greenhouse gases (e.g., Rogger et al. 2024, and references therein). The pace of the post-LIP cooling process on Earth is largely controlled by plant migration, which affects the intensity of vegetation-enhanced weathering (Rogger et al. 2024). Other strong stresses on the biosphere include oceanic anoxia, ocean acidification, and toxic metal input (Ernst and Youbi 2017). However, it should be noted that some of the feedback mechanisms characteristic of plate tectonics may also operate in the squishy-lid tectonic regime, due to various regional-scale tectono-magmatic activities (Sizova et al. 2015; Lourenço et al. 2018) that can drive topographic changes and landscape and climate evolution. Whether this more limited set of mechanisms is sufficient to enable the emergence of life, and more importantly, to sustain and promote its long-term evolution, remains unclear.

### Importance of Coexisting Subaerial Land and Oceans

As discussed in the previous section, many of the feedback mechanisms (e.g., weathering, erosion, landscape evolution, carbon cycle, climate amelioration etc.) are most efficient in the presence of coexisting subaerial land and oceans on a planetary surface. This is an extremely limiting condition from a planetary perspective (Stern and Gerya 2024, Spohn et al. (2026, this collection), and Guimond et al. (2026, this collection)). Life on Earth originated before 3.8 Ga (Schidlowski 1988; Mojzsis et al. 1996) and evolved in the oceans for more than 3 Gyr, although the presence of exposed subaerial land may also have been crucial for the origin and evolution of life (e.g., Retallack 2016; Korenaga 2021b). Thermodynamic considerations indicate that the absence of exposed land would have caused the complete hydrolysis of all polymers and metabolites (Martin 1998; Deamer and Weber 2010). In addition, some have suggested that life originated and evolved initially in hydrated regolith on land rather than in the oceans (Retallack 2016). On the other hand, there are at least three reasons why the evolution of life up to the point of metazoans must have occurred in water (Stern and Gerya 2024). First, seawater contains dissolved nutrients which can be easily absorbed through initially primitive and then increasingly sophisticated cell walls. Second, seawater protects organisms from deadly ultraviolet radiation. It was not until approximately 2.4 Gyr ago that photosynthetic cyanobacteria oxygenated the atmosphere of the Earth to form a protective stratospheric ozone layer, significantly reducing the flux of UV radiation reaching the surface of the Earth (Cooke et al. 2022). Third, single-cell life had to evolve before more complex multicellular organisms could evolve from them. This had to occur in water due to the structural support that seawater provides. This structural support is also needed for early soft-bodied multicellular organisms. These organisms could not thrive on land until subsequent evolution and natural selection developed hard, strong exo- and endoskeletons that allowed creatures to contend with gravity.

Although primitive life evolved in the sea, the plate tectonics evolutionary pump seems to work more efficiently on land (Pellissier et al. 2018; Liu et al. 2024). First, changing landscapes provide more varied habitats than seascapes, which is necessary to accelerate the evolution and diversification of complex species. Regions with high tectonic complexity, predominantly located at the confluence of major lithospheric plates such as the circum-Mediterranean, Mesoamerica, Madagascar and South East Asia, are especially favorable sites for allopatric speciation and the emergence of new land species across straits. This correlation is much less pronounced for marine species, mainly because the marine realm is more permeable to the movement of organisms (Palumbi 1994; Pellissier et al. 2018). Furthermore, dry land stimulated the adaptations necessary for survival in harsh terrestrial environments (Garwood and Edgecombe 2011): water retention, specialized gas exchange structures, reproduction by predominantly internal fertilization, locomotion in the absence of structural support, adapted eyes and newly developed senses. These conditions stimulated the development of various animal appendages adapted for locomotion, feeding, manipulation, and other functions (Panganiban et al. 1997; Shield et al. 2021), and helped to adapt the eyes and other senses and the central nervous system to the new environment and functions. The resulting sophisticated bioassets enabled increasingly intelligent creatures to populate and explore the highly variable terrestrial environments. This was one (but not the only) prerequisite for increasingly developing and transferring various experiences (i.e., knowledge and information) about these environments within biological populations. This could potentially (but not necessarily) result in the beginning of abstract thinking, leading to the development of intelligence, technology, and civilization (Stern and Gerya 2024).

## Conclusions and Outlook

### A Unifying Heat-Engine Framework

In this article, we have attempted to frame rocky planets as heat engines, in which internal heat sources lead to various import dynamics that mold the evolution of a planet. We aimed at providing a unifying framework that connects a wide range of phenomena across rocky bodies in the Solar System and beyond, including mantle convection, core convection, tectonic regimes, magmatism, crust formation, volatile cycling, magnetic field generation, and, ultimately, planetary habitability. While the details of many of these are complex and complicated by uncertainties, for example, in mineral physics, rheology, and geochemistry, the heat-engine framework emphasizes first-order energy balances and feedbacks that are robust across planetary scales and can be readily applied to rocky exoplanets.

A key point in this review is that mantle convection should be ubiquitous among rocky planets. Given the intense heating associated with accretion and differentiation, supplemented by long-lived radiogenic heating and, in some cases, tidal heating, the Rayleigh numbers of silicate mantles are expected to exceed critical values by orders of magnitude for most rocky planets. The fundamental question is therefore not whether rocky planets convect, but how convection is expressed at the surface and how efficiently the planet’s heat engine operates over time. The surface expression of interior dynamics can be referred to as a tectonic regime and acts as a regulator of planetary cooling, compositional evolution, and interior–surface coupling.

Within our Solar System, a striking diversity of tectonic (and magmatic) regimes are observed, from Earth’s plate tectonics to Venus’ likely episodic or plutonic-squishy lid behavior, Mars’ stagnant lid with localized volcanism, Mercury’s thin-mantle evolution, and Io’s tidally driven heat-pipe regime. This diversity demonstrates that plate tectonics is only one regime within several and is not an inevitable outcome of mantle convection. Numerical and theoretical studies further show that multiple tectonic states may be dynamically stable for the same bulk planetary parameters, implying a strong dependence on the history of the planet. Early events, such as magma-ocean solidification style, volatile loss or retention, atmosphere and impact history, and early crust formation, can permanently steer a planet toward one evolutionary pathway or another (e.g., Earth vs. Venus).

Crust formation and redistribution are central processes in the planetary heat engine. Melting not only transports heat efficiently via latent heat and magmatism but also redistributes incompatible elements, including radiogenic heat sources, into the crust. The resulting crustal layer can either enhance cooling during periods of intense volcanism or progressively insulate the mantle at later stages, slowing secular cooling. The balance between extrusion and intrusion exerts strong control on lithospheric strength, lid thickness, and tectonic style, particularly for planets lacking sustained subduction. In this sense, crust is not a passive byproduct of mantle convection, but an active regulator of planetary evolution.

The operation of the planetary heat engine also governs the generation and longevity of magnetic fields. Core dynamos depend on the rate at which heat and/or buoyancy is extracted from the core, which in turn is controlled by mantle convection and the active tectonic regime. Efficient cooling associated with mobile-lid or plate-tectonic behavior favors long-lived dynamos, whereas stagnant-lid planets tend to shut down thermal dynamos early and may only host transient or chemically driven dynamos later (if at all). The diversity of magnetic field histories inferred for Earth, Mars, and Mercury shows the coupling between mantle and core evolution and highlights how magnetic fields can inform us about the interior of a planet.

Finally, we attempted to show how the interior dynamics of a planet is deeply intertwined with habitability and bio-geodynamics. The tectonic regime influences volatile cycling, atmospheric composition, climate stability, nutrient delivery, and the availability of coexisting oceans and subaerial land. Plate tectonics, in particular, appears to maximize the diversity and efficiency of these feedbacks on Earth, but it is not the only conceivable pathway to surface habitability. Alternative regimes, such as the heat-pipe or plutonic–squishy lid, may sustain active volatile cycling and habitable conditions under certain circumstances, but future work is needed to clarify this, especially for planets with different masses, compositions, or stellar environments.

### Implications for Exoplanets

When extending insights from the Solar System to exoplanets, an important inference is that interior dynamics cannot be inferred from bulk properties alone. Planetary mass and radius provide constraints on mean density and broad interior structure, but they do not uniquely determine the tectonic regime, magmatic style, or magnetic history. Factors such as volatile inventory, surface temperature, distance to the star, and early magma ocean evolution complicate matters. As a result, Earth-like mass and radius do not imply Earth-like geodynamics.

Nevertheless, the heat-engine framework offers some guidance for interpreting (sparse) exoplanet data. For example, planets subjected to strong tidal forcing are natural candidates for enhanced internal heating, potentially leading to heat-pipe or magma-dominated regimes. Planets with moderate surface temperatures and sufficient volatile retention are more likely to experience episodic or sustained lithospheric recycling. The size of the planet is another source of uncertainty: in Super-Earths, increased internal pressures lead to different phase changes, melting relations, and viscosity profiles in ways that can either promote or suppress efficient cooling and dynamo action, depending on incompletely understood pressure dependencies.

### Outlook and Directions for Future Research

Progress in understanding rocky planets will depend on advances in several disciplines. We highlight some of them next.

1. **Improved constraints on material properties at extreme conditions are essential.** Many known rocky exoplanets are substantially more massive than Earth. Uncertainties in modeling super-Earth interiors and deep mantles arise largely from poorly constrained rheological laws, melting relations, and transport properties at pressures far exceeding those in Earth’s mantle. Advances in experimental, theoretical, and computational mineral physics, particularly in constraining viscosity, thermal conductivity, and the influence of volatiles and light elements, will directly improve predictions of convective vigor, tectonic regimes, and magnetic field evolution, amongst others.
2. **Treat the coupling between the interior, surface, and atmosphere more self-consistently.** Traditional models often prescribe surface temperature, tectonic regime, or volatile cycling as independent boundary conditions. However, as emphasized throughout this article, these components are dynamically coupled through strong feedbacks. Next-generation models that integrate mantle convection, crustal production, magnetic field evolution, volatile degassing and ingassing, climate evolution, and atmospheric escape will be essential for quantifying long-term habitability and improving the interpretation of exoplanet observations.
3. **Better understand transitions between tectonic regimes.** Many rocky planets may not have experienced a single tectonic regime throughout their evolutions but instead transition between different tectonic regimes as internal and surface conditions evolve. Understanding the triggers, timescales, and observability of such transitions is key to reconstructing planetary histories and to identifying signatures that might be detectable remotely, such as different types of atmospheres built by different tectonic regimes.
4. **Constrain whether habitability can arise and be sustained under tectonic regimes other than plate tectonics.** Although life on Earth possibly emerged prior to the onset of modern plate tectonics, plate tectonics appears to be a requirement for the long-term maintenance of habitable surface conditions and possibly for the development of complex life. Recent studies exploring alternative tectonic regimes suggest that at least some of the necessary conditions for habitability could be met without classical plate tectonics. Future work should aim to quantify the extent to which such regimes can enable the emergence of habitable conditions, sustain long-term habitability, and support the development of complex life.
5. **Exoplanet observations will increasingly inform geodynamics (and vice-versa).** While direct observation of tectonics is not yet feasible, indirect constraints are emerging. Atmospheric compositions can reflect long-term volcanic outgassing and volatile recycling, and thus a link to tectonic regimes. Measurements of planetary ages, stellar irradiation, and orbital patterns can constrain thermal and tidal evolution. Future detections of exoplanet magnetic fields, though challenging, would provide powerful information on core dynamics and interior cooling. Interpreting these data will require close integration between observational astronomy and planetary geophysics and geology.
6. **A comparative and population-based perspective is important.** The Solar System provides samples of only a tiny fraction of possible planetary outcomes. Exoplanet discoveries expand the parameter space dramatically, revealing planets with masses, temperatures, and orbital environments absent in our own system. Rather than asking whether Earth is typical or unique, future work should aim to map where Earth lies within a multidimensional landscape of planetary heat engines, and which combinations of parameters favor particular evolutionary trajectories.

### Closing Remarks

The central message of this article is that interior dynamics is fundamental to understanding a planet’s present state. Viewing rocky planets as heat engines provides a framework for better interpreting how interior dynamics shape surfaces, atmospheres, magnetic fields, and habitability. The remarkable diversity of rocky bodies in the Solar System already demonstrates that small differences in initial conditions or boundary conditions can lead to divergent outcomes. In this sense, the study of rocky planets lies at an exciting place of intersection of geophysics, astronomy, and astrobiology. By combining insights from the Solar System with rapidly expanding exoplanet observations and by grounding interpretation in the physics of planetary heat engines, we move closer to understanding not only how planets function, but how common or rare pathways leading to Earth-like worlds and life may be in our galaxy. We hope that this article contributes to this growing body of knowledge.

## Footnotes

1 Tidal interactions can result in decreasing orbital distances and increasing eccentricities and obliquities. But these are then not immediately related to tidal dissipation.
